# Type I Interferon Induced by *Streptococcus suis* Serotype 2 is Strain-Dependent and May Be Beneficial for Host Survival

**DOI:** 10.3389/fimmu.2017.01039

**Published:** 2017-08-28

**Authors:** Jean-Philippe Auger, Agustina Santinón, David Roy, Karen Mossman, Jianguo Xu, Mariela Segura, Marcelo Gottschalk

**Affiliations:** ^1^Research Group on Infectious Diseases in Production Animals (GREMIP), Swine and Poultry Infectious Diseases Research Center (CRIPA), Faculty of Veterinary Medicine, Department of Pathology and Microbiology, University of Montreal, Saint-Hyacinthe, QC, Canada; ^2^Department of Biochemistry and Biomedical Sciences, McMaster University, Hamilton, ON, Canada; ^3^Collaborative Innovation Center for Diagnosis and Treatment of Infectious Diseases, National Institute for Communicable Disease Control and Prevention, Chinese Center for Disease Control and Prevention, Beijing, China

**Keywords:** *Streptococcus suis* serotype 2, type I interferon, dendritic cells, inflammation, virulence

## Abstract

*Streptococcus suis* serotype 2 is an important porcine bacterial pathogen and emerging zoonotic agent mainly responsible for sudden death, septic shock, and meningitis, with exacerbated inflammation being a hallmark of the infection. However, serotype 2 strains are genotypically and phenotypically heterogeneous, being composed of a multitude of sequence types (STs) whose virulence greatly varies: the virulent ST1 (Eurasia), highly virulent ST7 (responsible for the human outbreaks in China), and intermediate virulent ST25 (North America) are the most important worldwide. Even though type I interferons (IFNs) are traditionally associated with important antiviral functions, recent studies have demonstrated that they may also play an important role during infections with extracellular bacteria. Upregulation of IFN-β levels was previously observed in mice following infection with this pathogen. Consequently, the implication of IFN-β in the *S. suis* serotype 2 pathogenesis, which has always been considered a strict extracellular bacterium, was evaluated using strains of varying virulence. This study demonstrates that intermediate virulent strains are significantly more susceptible to phagocytosis than virulent strains. Hence, subsequent localization of these strains within the phagosome results in recognition of bacterial nucleic acids by Toll-like receptors 7 and 9, leading to activation of the interferon regulatory factors 1, 3, and 7 and production of IFN-β. Type I IFN, whose implication depends on the virulence level of the *S. suis* strain, is involved in host defense by participating in the modulation of systemic inflammation, which is responsible for the clearance of blood bacterial burden. As such, when induced by intermediate, and to a lesser extent, virulent *S. suis* strains, type I IFN plays a beneficial role in host survival. The highly virulent ST7 strain, however, hastily induces a septic shock that cannot be controlled by type I IFN, leading to rapid death of the host. A better understanding of the underlying mechanisms involved in the control of inflammation and subsequent bacterial burden could help to develop control measures for this important porcine and zoonotic agent.

## Introduction

*Streptococcus suis* is an important porcine bacterial pathogen and emerging zoonotic agent mainly responsible for sudden death (pigs), septic shock (humans), and meningitis (both species) (1). Of the different described serotypes, an important taxonomical classification for this pathogen based on the presence of the capsular polysaccharide (CPS) or its respective genes, serotype 2 is regarded as not only the most widespread worldwide, but also the most virulent, responsible for the majority of porcine and human cases of infection (2). However, serotype 2 strains are genotypically and phenotypically heterogeneous, resulting in them being classified into a multitude of sequence types (STs), as determined by multilocus sequence typing, whose distribution greatly varies worldwide (2). As such, the various *S. suis* strains belonging to serotype 2 are grouped into different STs based on shared genetic similarities that better explain the evolutionary divergence of this pathogen. Virulence of the most important STs (ST1, ST7, and ST25) has been evaluated using mouse models of infection (3, 4). Indeed, the ST7 strain responsible for the human outbreaks of 1998 and 2005 in China is highly virulent whereas European ST1 strains are virulent; on the other hand, ST25 strains, typically recovered in North America, are of intermediate virulence (3, 5).

Of the various virulence factors described for *S. suis*, the CPS, suilysin (SLY), and cell wall modifications have been demonstrated to play important roles (6, 7). Indeed, the CPS, which is antigenically identical for all serotype 2 strains, is a critical factor implicated in a multitude of functions, most importantly in resistance to phagocytosis by innate immune cells (8–12); its presence also masks bacterial surface proteins responsible for host cell activation (12, 13). Meanwhile, the SLY, a cholesterol-dependent cytolysin similar to the pneumolysin of *Streptococcus pneumoniae*, is responsible for causing cell toxicity and inducing pro-inflammatory cytokines (12, 14). This toxin is present in serotype 2 ST1 and ST7 strains, but not in ST25 strains (14, 15). Cell wall modifications, such as the D-alanylation of the lipoteichoic acid and N-deacetylation of the peptidoglycan, of a ST1 strain were shown to interfere with host defense and to be partially responsible for cell activation (12, 16, 17). Finally, several cell wall-associated proteins, mainly reported for ST1 and ST7 strains, have also been described as critical virulence factors, though many of these remain controversial in the literature (7).

Recognition of *S. suis* by innate immune cells involves a multitude of membrane-associated and cytoplasmic receptors (6, 18). Of these, the Toll-like receptor (TLR) pathway is implicated in recognition of *S. suis* by phagocytic cells, including dendritic cells (DCs) and macrophages (19), while recognition by other cells types, such as epithelial cells, remains unknown. Abrogation of MyD88, the adaptor protein central to the TLR pathway, results in near complete lack of pro-inflammatory cytokine production *in vitro* following infection with *S. suis* (13, 20). Furthermore, being a classical extracellular pathogen, recognition of *S. suis* has been mostly associated with surface TLRs, where TLR2, in cooperation with TLR6, plays a predominant role (13, 20, 21). Pathogen recognition by TLRs classically results in the production of pro-inflammatory cytokines *via* the NF-κB or interferon (IFN) pathways (19). Pathways involved in activation of NF-κB by *S. suis* have been somewhat described in recent years (20, 22), while those regarding the IFN pathways are less known, having mainly focused on type II IFN (3, 23). Meanwhile, the capacity of *S. suis* to induce type III IFN was only evaluated once, *in vitro*, and for IFNλ1 only, with negative results obtained (24). Nonetheless, it was recently demonstrated that expression levels of the type I IFN, IFN-β, but not those of IFN-α, are upregulated *in vivo* following infection with *S. suis* serotype 2 (3). Moreover, this upregulation of IFN-β was significantly higher in mice infected with an intermediate virulent ST25 strain than in those infected with either a virulent ST1 strain or the highly virulent ST7 strain responsible for the human outbreaks (3). However, no other study has addressed the production of type I IFN or its role during the *S. suis* serotype 2 infection.

Type I IFN regroups various members of the IFN family of which IFN-α, composed of 16 different subtypes, and IFN-β, its most potent member, are the best characterized (25). Classical production of these cytokines is the result of endosomal TLR (TLR3, TLR7, and TLR9 in mice) activation, which leads to phosphorylation and translocation of interferon regulatory factors (IRFs) to the nucleus (26). Though IRF1 and IRF3/IRF7 are usually associated with type II and type I IFN, respectively, all three can result in transcription of type I IFNs (25, 26). Following their production, both IFN-α and IFN-β bind a common heterodimeric receptor, the IFN-α/β receptor (IFNAR) (26). Binding to this receptor activates the Janus kinase (JAK)–signal transducer and activator of transcription (STAT) pathway, resulting in transcription of various genes associated with host defense and modulation of the inflammatory response (26).

Even though type I IFNs are traditionally associated with important antiviral functions, recent studies have demonstrated that they may also play an important role, particularly for IFN-β, during bacterial infections, including pathogenic streptococci (26, 27). However, their role, whether beneficial or detrimental, may depend on the bacterial species and/or infection model (28–32). As aforementioned, little information is available regarding type I IFN, and more specifically IFN-β, during the *S. suis* infection, which is considered a strict extracellular pathogen. Consequently, its implication in the *S. suis* serotype 2 pathogenesis was evaluated using strains of varying virulence. Herein, we demonstrated that following phagocytosis by DCs, to which intermediate virulence strains are more susceptible, *S. suis* is located within the phagosome where bacterial nucleic acids are recognized by TLR7 and TLR9, leading to activation of IRF1, IRF3, and IRF7 and production of IFN-β. When induced by intermediate, and to a lesser extent, virulent *S. suis* strains, but not by a highly virulent strain, type I IFN plays a beneficial role, being involved in the control of blood bacterial burden *via* modulation of systemic inflammation.

## Materials and Methods

### Ethics Statement

This study was carried out in accordance with the recommendations of the guidelines and policies of the Canadian Council on Animal Care and the principles set forth in the Guide for the Care and Use of Laboratory Animals. The protocols and procedures were approved by the Animal Welfare Committee of the University of Montreal (protocol number rech-1570).

### Endotoxin-Free Conditions

Endotoxin (lipopolysaccharide)-free material and solutions were used for bacterial and cell culture work throughout this study.

### *S. suis* Serotype 2 Strains and Growth Conditions

The different well-encapsulated *S. suis* serotype 2 strains, belonging to the most important STs (ST1, ST7, and ST25), and isogenic mutants used in this study are listed in Table 1. A highly virulent ST7 strain, isolated during the 2005 human outbreak in China (SC84) (33), a virulent prototype European ST1 strain (P1/7), and an intermediate virulent North American ST25 strain (89-1591) (3) were used throughout this study. Isogenic mutants derived from P1/7 (ST1) or a genotypically and phenotypically similar strain, 31533 (ST1), were also included in this study. For comparison purposes, two additional intermediate virulent ST25 strains (91-1804 and LPH4) were used in selected experiments. Virulence of the wild-type strains was previously reported (3, 4). *S. suis* strains were grown in Todd Hewitt broth (THB; Becton Dickinson, Mississauga, ON, Canada) as previously described (10), diluted in culture medium before experiments, and the number of colony-forming units (CFUs)/mL in the final suspension determined by plating on THB agar.

**Table 1:** *Streptococcus suis* serotype 2 strains used in this study.

Strain	General characteristics	Reference
P1/7	Virulent European ST1 strain isolated from a case of pig meningitis in the United Kingdom	(34)
P1/7Δ*cpsF*	Isogenic non-encapsulated mutant derived from P1/7; in frame deletion of *cpsF* gene	(12)
31533	Virulent European ST1 strain isolated from a case of pig meningitis in France	(35)
31533Δ*sly*	Isogenic suilysin-deficient mutant derived from 31533; in frame deletion of *sly* gene	(36)
31533Δ*dltA*	Isogenic d-alanylation of lipoteichoic acid-deficient mutant derived from 31533; in frame deletion of *dltA* gene	(17)
31533Δ*pgdA*	Isogenic *N*-deacetylation of peptidoglycan-deficient mutant derived from 31533; in frame deletion of *pgdA* gene	(16)
SC84	Highly virulent ST7 strain isolated from a case of human streptococcal toxic shock-like syndrome during the 2005 outbreak in China	(33)
89-1591	Intermediate virulent North American ST25 strain isolated from a case of pig sepsis in Canada	(37)
91-1804	Intermediate virulent North American ST25 strain isolated from a case of human endocarditis in Canada	(38)
LPH4	Intermediate virulent Asian ST25 strain isolated from a case of human sepsis in Thailand	(39)

### Mice

MyD88^−/−^ [B6.129P2(SJL)-*MyD88^tm1.Defr^*/J], TLR2^−/−^ (B6.129-*Tlr2^tmKir^*/J), TLR3^−/−^ (B6.129S1-*Tlr3^tm1Flv^*/J), TLR4^−/−^ (B6.B10ScN-*Tlr4^lps-del^*/JthJ), TLR7^−/−^ (B6.129S1-*Tlr7^tm1Flv^*/J), TLR9^−/−^ (C57BL/6J-*Tlr9^M7Btlr^*/Mmjax), IRF1^−/−^ (B6.129S2-*Irf1^tm1Mak^*/J), IRF3^−/−^ (40), IRF7^−/−^ (41), and IFNAR1^−/−^ (B6.129S2-*Ifnar1^tm1Agt^*/Mmjax) mice on C57BL/6 background were housed under specific pathogen-free conditions alongside their wild-type counterparts (C57BL/6J). Mice were purchased from Jackson Research Laboratories (Bar Harbor, ME, USA), with the exception of IRF3^−/−^ and IRF7^−/−^ mice, which were provided by Dr. K. Mossman.

### Generation of Bone Marrow-Derived DCs and Macrophages

The femur and tibia of wild-type and knock-out mice were used to generate bone marrow-derived DCs and macrophages, as previously described (12, 42). Briefly, hematopoietic bone marrow cells were cultured in RPMI-1640 medium supplemented with 5% (DCs) or 10% (macrophages) heat-inactivated fetal bovine serum, 10 mM HEPES, 2 mM l-glutamine, and 50 µM 2-mercaptoethanol (Gibco, Burlington, ON, Canada). Complete medium was complemented with 20% granulocyte macrophage-colony stimulating factor from mouse-transfected Ag8653 cells for DCs (42) or L929 cell-derived macrophage-colony stimulating factor for macrophages (43). Cell purity was at least 85% CD11c^+^ and F4/80^+^ cells for DCs and macrophages, respectively (12).

### *S. suis* Infection of DCs and Macrophages

Cells were resuspended at 1 × 10^6^ cells/mL in complete medium and stimulated with the different *S. suis* serotype 2 strains listed in Table 1 (10^6^ CFU/mL; initial multiplicity of infection = 1). The conditions used were based on those previously published (12, 20). Cells were harvested in TRIzol (Invitrogen, Burlington, ON, Canada) for mRNA expression 3, 6, or 12 h following infection, and supernatants collected for cytokine measurement 16 h [tumor necrosis factor (TNF), interleukin (IL)-6, IL-12p70, C–C motif chemokine ligand (CCL) 2, CCL3, and C–X–C motif chemokine ligand (CXCL) 1] or 24 h (IFN-β) post-infection (p.i.). These mediators were selected on the basis that DCs and macrophages secrete important levels of these cytokines and chemokines following infection by *S. suis* and that levels of these mediators are elevated following infection *in vivo* (12, 13, 20). Non-infected cells served as negative controls. For neutralization of TLR9, DCs were pretreated with 5 µM ODN2088 (TLR9 inhibitor; InvivoGen, Burlington, ON, Canada) or 5 µM ODN2088-control (Ctrl) for 1 h prior to infection with *S. suis*. Different TLR ligands were used to stimulate cells as positive controls: 1 µg/mL PAM3CSK4 (TLR1/2; InvivoGen), 1 µg/mL FSL-1 (TLR2/6; InvivoGen), 10 µg/mL poly(I:C) (TLR3; Novus Biologicals, Littleton, CO, USA), 100 ng/mL ultrapure *Escherichia coli* lipopolysaccharide (TLR4; InvivoGen), 5 µg/mL imiquimod (TLR7; Novus Biologicals), and 1 µM CpG ODN1826 (TLR9; InvivoGen).

### Determination of Cell mRNA Expression by Quantitative RT-PCR

Cell mRNA was extracted according to the manufacturer’s instructions (TRIzol) and cDNA generated using the Quantitect cDNA Synthesis Kit (Qiagen, Mississauga, ON, Canada). Real-time qPCR was performed on the CFX-96 Touch Rapid Thermal Cycler System (Bio-Rad, Hercules, CA, USA) using 250 nM of primers (Integrated DNA technologies, Coralville, IA, USA) and the SsoFast Evagreen Supermix Kit (Bio-Rad). The cycling conditions were 3 min of polymerase activation at 98°C, followed by 40 cycles at 98°C for 2 s and 57°C for 5 s. Melting curves were generated after each run to confirm the presence of a single PCR product. The sequences of primers used in this study are shown in Table S1 in Supplementary Material and were verified to have reaction efficiencies between 90% and 110%. The reference genes *Atp5b* and *Gapdh*, determined to be the most stably expressed using the algorithm geNorm, were used to normalize data. Fold changes in gene expression were calculated using the quantification cycle threshold (Cq) method using the CFX software manager v.3.0 (Bio-Rad). Samples from mock-infected cells served as calibrators.

### Cytokine Quantification in Cell Culture Supernatants

Levels of IFN-β, TNF, IL-6, IL-12p70, CCL2 (MCP-1), CCL3 (MIP-1α), and CXCL1 (KC) in cell culture supernatants were measured by sandwich ELISA using pair-matched antibodies from BioLegend (Burlington, ON, Canada) for IFN-β and from R&D Systems (Minneapolis, MN, USA) for the other cytokines, according to the manufacturer’s recommendations.

### Phagocytosis Assays

Cells were infected with the different *S. suis* strains and phagocytosis was left to proceed for different times (0.5–4 h) at 37°C with 5% CO_2_. Multiplicity of infection and assay conditions were chosen based on previous studies regarding the kinetics of *S. suis* phagocytosis by DCs (12). After incubation, penicillin G (5 mg/mL; Sigma-Aldrich, Oakville, ON, Canada) and gentamicin (100 mg/mL; Gibco) were directly added to the wells for 1 h to kill extracellular bacteria. Supernatant controls were taken in every test to confirm that extracellular bacteria were efficiently killed by the antibiotics. After antibiotic treatment, cells were washed three times and sterile water added to lyse the cells. Where required, cells were pretreated with either 5 µM cytochalasin D (Santa Cruz Biotech, Dallas, TX, USA), 8 µM dynasore (Sigma-Aldrich), 1 µM bafilomycin A1 (Santa Cruz Biotech) or their vehicle, DMSO (Sigma-Aldrich), for 45 min prior to infection with bacteria, and phagocytosis allowed to proceed for 2 h. The number of CFU recovered per well was determined by plating viable intracellular bacteria on THB agar.

### *S. suis* DNA and RNA Preparation and Transfection of Cells

For bacterial RNA and DNA isolation, bacterial cultures were grown to mid-log phase. Total RNA was extracted using the Aurum Total RNA Mini Kit (Bio-Rad) according to the manufacturer’s instructions, including treatment with DNase I. For DNA preparation, bacteria were harvested in 10 mM Tris, 1 mM EDTA, pH 8.0, and treated with 10% SDS and 20 mg/mL proteinase K (Sigma-Aldrich) for 1 h at 37°C. DNA was isolated using phenol/chloroform/isoamyl alcohol (Sigma-Aldrich) (28). After isolation, bacterial DNA was treated with 10 mg/mL RNase A (Roche, Mississauga, ON, Canada) for 30 min at 37°C. DCs were transfected with 1 µg of RNA or DNA complexed or not with DOTAP liposomal transfection agent (Sigma-Aldrich) as described for transfection of bacterial extracts (28, 44).

### *S. suis* Serotype 2 Mouse Model of Infection

Six-week-old wild-type C57BL/6 and IFNAR^−/−^ mice were used. Mice were acclimatized to standard laboratory conditions with unlimited access to water and rodent chow (45). These studies were carried out in strict accordance with the recommendations of and approved by the University of Montreal Animal Welfare Committee guidelines and policies, including euthanasia to minimize animal suffering, applied throughout this study when animals were seriously affected since mortality was not an end point measurement. The different *S. suis* serotype 2 strains, or the vehicle solution (sterile THB), were administered at a dose of 1 × 10^7^ CFU by intraperitoneal inoculation to groups of 15 mice for survival and blood bacterial burden. Mice were monitored at least three times daily until 72 h p.i. and twice thereafter until 14 days p.i. Blood bacterial burden was assessed 12 and 48 h p.i. by collecting 5 µL of blood from the caudal vein, appropriately diluting and plating on THB agar as described above. Blood bacterial burden was also measured prior to euthanasia.

### Measurement of Plasma (Systemic) Pro-inflammatory Cytokine Levels

Eight wild-type and IFNAR^−/−^ mice per group were infected with each strain as described above and the blood collected 12 h p.i. by intracardiac puncture following euthanasia and stabilization with EDTA (Sigma-Aldrich) as previously described (3, 4). Plasma supernatants were collected following centrifugation at 10,000 × *g* for 10 min at 4°C and stored at −80°C. Plasmatic concentrations of TNF, IL-6, IL-12p70, CCL2, CCL3, and CXCL1 were measured using a custom-made cytokine Bio-Plex Pro™ assay (Bio-Rad) according to the manufacturer’s instructions. Acquisition was performed on the MAGPIX platform (Luminex^®^) and data analyzed using the Bio-Plex Manager 6.1 software (Bio-Rad).

### Transmission Electron Microscopy

Unless otherwise indicated, chemicals were purchased from Sigma-Aldrich. Bacteria were grown to mid-logarithmic phase and washed in 0.1 M cacodylate buffer, pH 7.3 (Canemco & Marivac, Canton de Gore, QC, Canada). The CPS was stabilized using specific antibodies as previously described (46). Anti-*S. suis* serotype 2 rabbit serum, produced as previously described (47), was used to gently resuspend bacteria. Next, cells were immobilized in 4% (w/v) agar in 0.1 M cacodylate buffer, pH 7.3. Pre-fixation was performed by adding 0.1 M cacodylate buffer, pH 7.3, containing 0.5% (v/v) glutaraldehyde, and 0.15% (w/v) ruthenium red for 30 min. Fixation was performed for 2 h at room temperature with 0.1 M cacodylate buffer, pH 7.3, containing 5% (v/v) glutaraldehyde, and 0.05% (w/v) ruthenium red. Post-fixation was carried out with 2% (v/v) osmium tetroxide in water overnight at 4°C. Samples were washed with water every 20 min for 2 h to remove osmium tetroxide and dehydrated in an increasing graded series of acetone. Specimens were then washed twice in propylene oxide and embedded in Spurr low-viscosity resin (Electron Microscopy Sciences, Hatfield, PA, USA). Thin sections were post-stained with uranyl acetate and lead citrate and examined using a transmission electron microscope at 80 kV (Hitachi model HT7770, Chiyoda, Tokyo, Japan).

### Statistical Analyses

Normality of data was verified using the Shapiro–Wilk test. Accordingly, parametric (unpaired *t*-test and one-way ANOVA) or non-parametric tests (Mann–Whitney rank sum test and one-way ANOVA on ranks), where appropriate, were performed to evaluate statistical differences between groups. Log-rank (Mantel–Cox) tests were used to compare survival between wild-type and IFNAR^−/−^ mice. Each test was repeated in at least three independent experiments. *p* < 0.05 and *p* < 0.01 values were considered as statistically significant and highly significant, respectively.

## Results

### Strain- and Cell Type-Dependent Induction of IFN-β by *S. suis* Serotype 2

**Figure 1:**
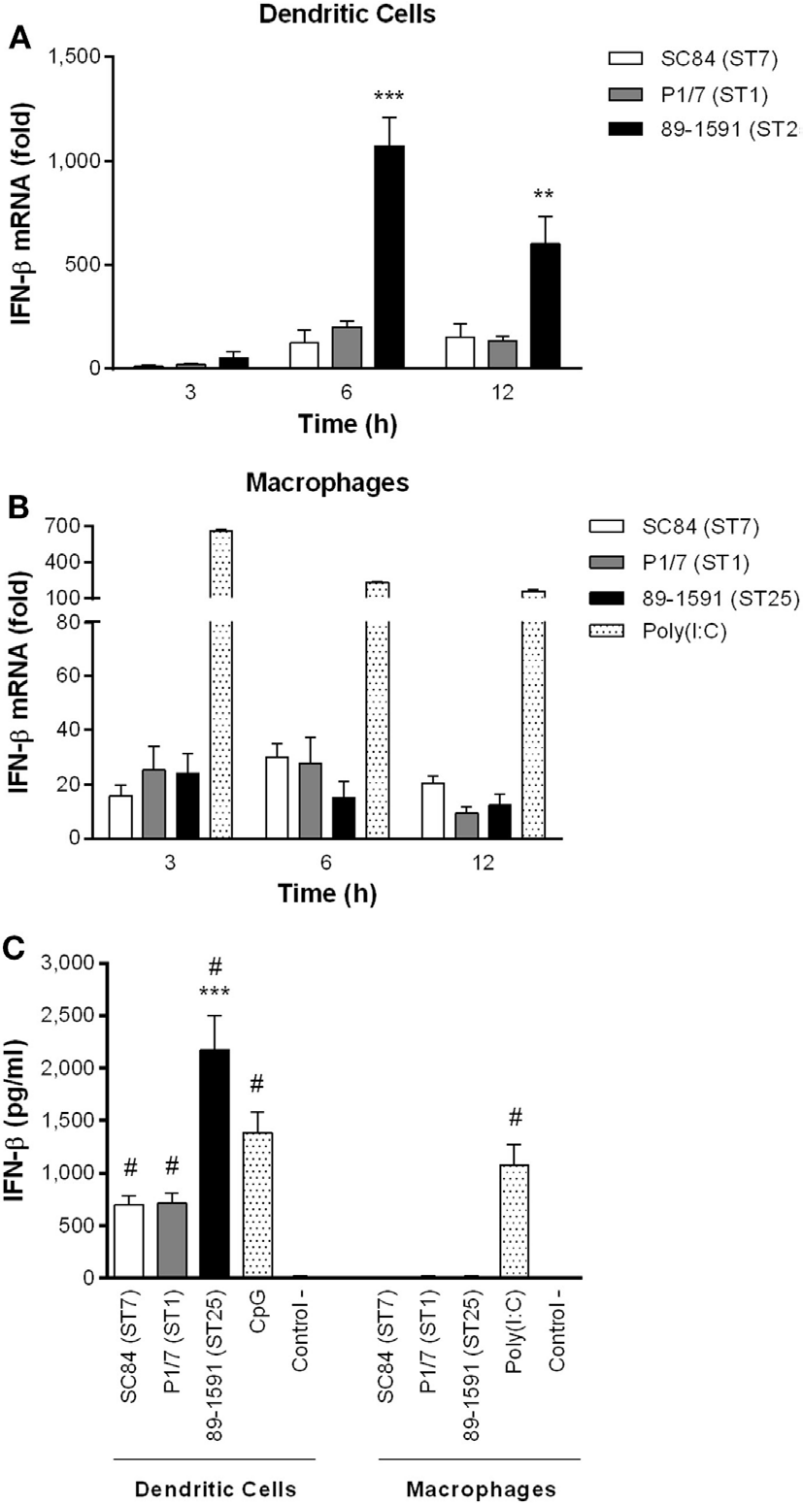
Dendritic cells (DCs) produce higher levels of interferon (IFN)-β than macrophages following infection with *Streptococcus suis* serotype 2. IFN-β mRNA expression kinetics, measured by quantitative RT-PCR, following infection of DCs **(A)** and macrophages **(B)**, with the highly virulent ST7 strain SC84, virulent ST1 strain P1/7, and intermediate virulent ST25 strain 89-1591. IFN-β protein production by DCs and macrophages was measured by ELISA 24 h following infection with the different *S. suis* strains **(C)**. Data represent the mean ± SEM (*n* = 4). ** (*p* < 0.01) and *** (*p* < 0.001) indicate a significant difference between 89-1591 and P1/7 or SC84; # (*p* < 0.001) between *S. suis* or CpG and the negative control (control −) for DCs or between poly(I:C) and negative control (control −) for macrophages.

Dendritic cells and macrophages have not only been demonstrated to be important for IFN-β production during bacterial infections, but were also shown to produce high levels of other pro-inflammatory cytokines following infection with *S. suis* (12, 13, 20, 48). Consequently, the capacity of these cells to produce IFN-β following infection with three different *S. suis* serotype 2 strains (highly virulent ST7 strain SC84, virulent ST1 strain P1/7, and intermediate virulent ST25 strain 89-1591) was evaluated. This cytokine was chosen as a representative of type I IFN since *S. suis* was previously demonstrated to upregulate levels of IFN-β expression *in vivo*, but not those of IFN-α, during acute infection (3). Moreover, DCs and macrophages have been demonstrated to mainly produce IFN-β, but only low levels of IFN-α, following infection with pathogenic streptococci (28, 30, 44). As shown in Figure 1A, DCs expressed low levels of IFN-β 3 h after infection with *S. suis* (Figure 1A). However, expression levels quickly and significantly increased, peaking at 6 h (*p* < 0.05). Importantly, levels induced by the intermediate virulent ST25 strain 89-1591 were significantly higher (*p* < 0.001) than those induced by the two more virulent strains. These levels were not specific to 89-1591, since similar high levels were also obtained using two other intermediate virulent ST25 strains (Figure S1 in Supplementary Material). In contrast, IFN-β expression levels of *S. suis*-infected macrophages remained relatively low and unchanged, regardless of the incubation time and strain used (Figure 1B). Indeed, IFN-β expression levels by macrophages were significantly lower than those by DCs and this, for all three strains tested (*p* < 0.01). A clear induction of IFN-β expression was detected with the positive control, poly(I:C), indicating that the low response observed with *S. suis* was a consequence of the stimulus rather than the cells (Figure 1B).

In order to evaluate if these differences in expression between cell types were also observed at the protein level, IFN-β was measured in the supernatant of cells 24 h after infection by ELISA (Figure 1C). Indeed, IFN-β mRNA expression and protein production correlated. Results demonstrated that only DCs produce important protein levels of IFN-β following infection with *S. suis* serotype 2, which were significantly higher than control cells (*p* < 0.001) (Figure 1C). However, as with mRNA expression, the intermediate virulent strain 89-1591 induced significantly higher protein levels of IFN-β by DCs than the other two *S. suis* strains (*p* < 0.001). Meanwhile, macrophages produced significant levels of IFN-β when stimulated with poly(I:C) (*p* < 0.001), but not following *S. suis* infection, confirming results observed at the transcriptional (mRNA) level (Figure 1C). Based on these results, all subsequent experiments in this study were performed using DCs.

### The Presence of CPS Interferes with *S. suis*-Induced IFN-β Expression by DCs, While the SLY (When Present) Is Partially Responsible for Activation

**Figure 2:**
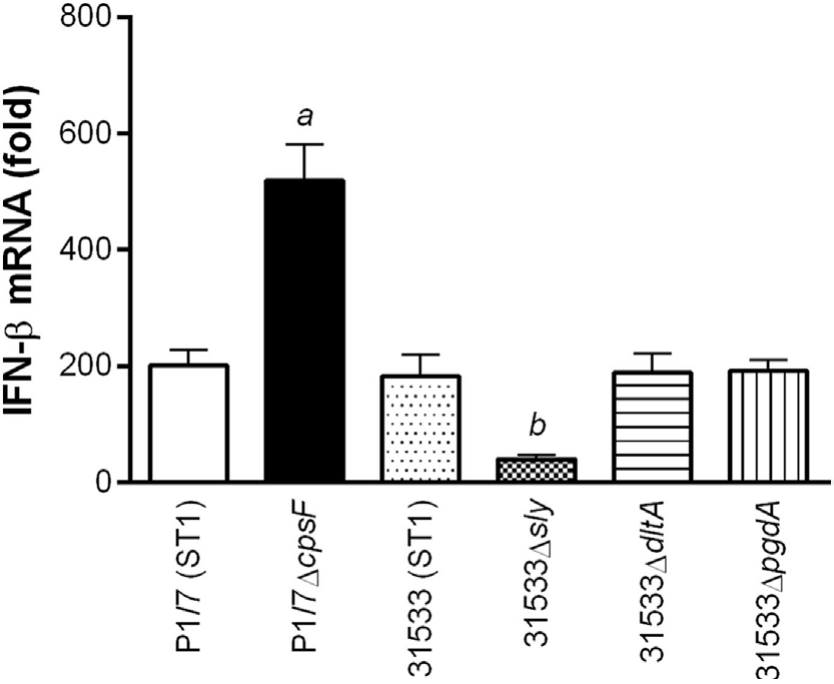
The presence of the capsular polysaccharide (CPS) interferes with *Streptococcus suis*-induced interferon (IFN)-β expression by dendritic cells (DCs), while the suilysin (SLY) is partially responsible for its activation. Role of the CPS, SLY, and cell wall modifications (D-alanylation of the lipoteichoic acid, Δ*dlta*, or *N*-deacetylation of the peptidoglycan, Δ*pgdA*) in IFN-β mRNA expression by DCs 6 h following infection with the wild-type or mutant *S. suis* strains. Data represent the mean ± SEM (*n* = 4). *a* (*p* < 0.001) indicates a significant difference between P1/7 and P1/7Δ*cpsF*; *b* (*p* < 0.01) between 31533 and 31533Δ*sly*.

Of the different described virulence factors for *S. suis* serotype 2, the presence of the CPS, SLY, and cell wall modifications has been reported to modulate and/or participate in cytokine production by DCs (12). Consequently, their role in the induction of IFN-β expression by DCs was evaluated using isogenic mutants (Figure 2). While the isogenic mutants used derived from two different wild-type strains (P1/7 and 31533), it was previously demonstrated that these two strains have a highly similar background (closely related virulent European ST1 strains) and induce similar levels of cytokine production by DCs (12). Absence of the CPS in a ST1 strain resulted in a significant increase of IFN-β expression by DCs (*p* < 0.001), suggesting that its presence interferes with cell activation. On the other hand, absence of the SLY, which is a pore-forming toxin present in ST1 and ST7 strains, resulted in a significant decrease of IFN-β expression (*p* < 0.01). Meanwhile, cell wall modifications (D-alanylation of the lipoteichoic acid and the *N*-acetylation of the peptidoglycan) did not modulate *S. suis*-induced IFN-β by DCs. As previously described for other cytokines, levels of IFN-β induced by the ST1 strain 31533 were very similar to those observed with the prototype strain P1/7.

### Recognition of *S. suis* by the TLR Pathway Is Required for IFN-β Induction in DCs

**Figure 3:**
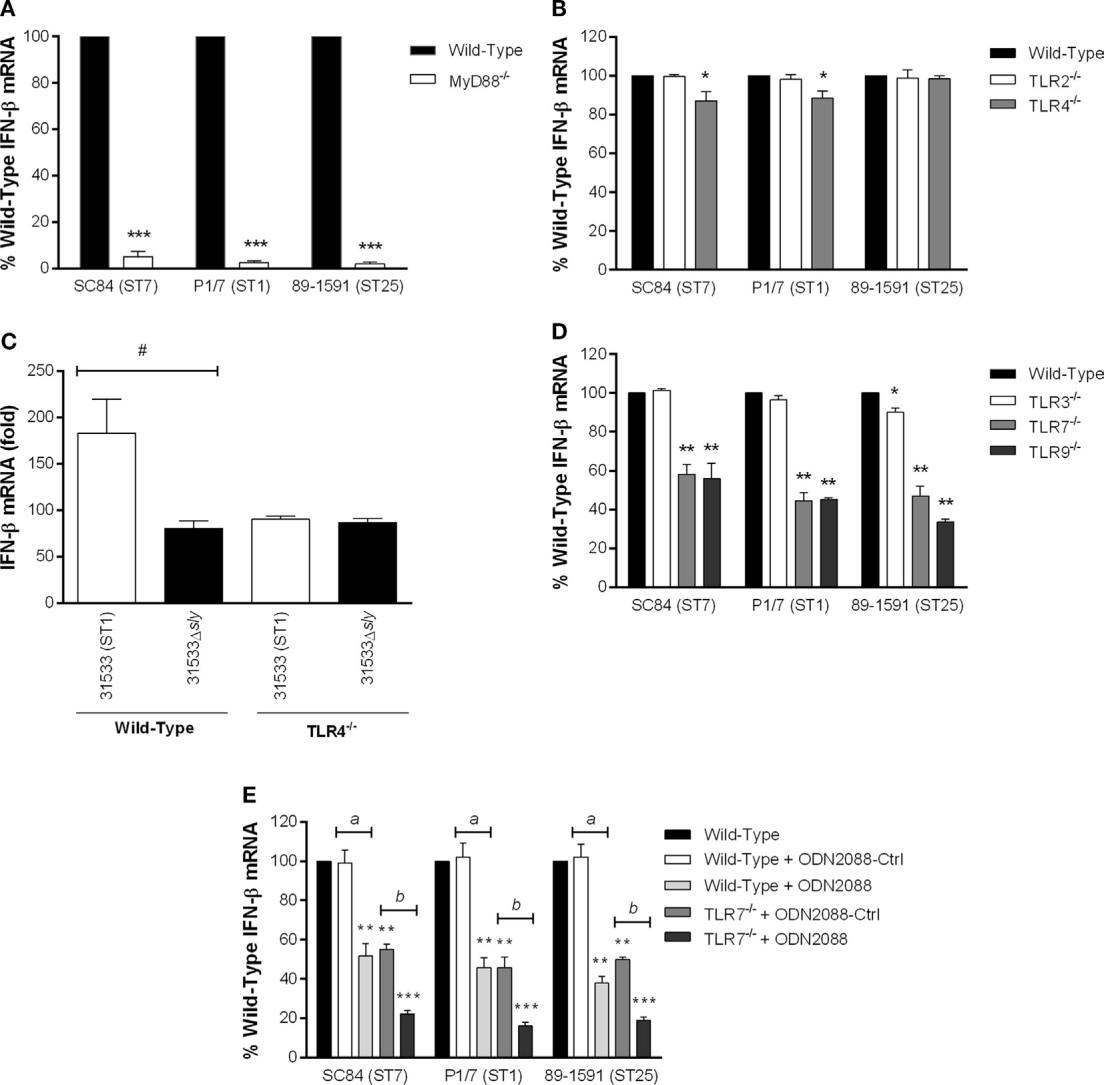
Recognition of *Streptococcus suis* by the Toll-like receptor (TLR) pathway is required for induction of interferon (IFN)-β expression by dendritic cells (DCs). IFN-β mRNA expression induced by the different *S. suis* strains 6 h following infection of DCs deficient for MyD88 **(A)**, TLR2, or TLR4 **(B)**, or for the endosomal TLR3, TLR7, or TLR9 **(D)**. The suilysin (SLY) is responsible for TLR4-dependent IFN-β expression by DCs **(C)**. The cooperative role of TLR7 and TLR9 was evaluated using wild-type or TLR7^−/−^ cells pretreated with the TLR9 antagonist ODN2088 or its control, ODN2088-Ctrl, resulting in TLR7^−/−^ cells non-responsive for TLR9 (dual deficiency) **(E)**. Data represent the mean ± SEM (*n* = 4). * (*p* < 0.05), ** (*p* < 0.01), or *** (*p* < 0.001) indicates a significant difference between expression by wild-type and deficient DCs; # (*p* < 0.01) between 31533 and 31533Δ*sly*; *a* (*p* < 0.01) between expression by wild-type DCs pretreated with ODN2088-Ctrl or ODN2088; *b* (*p* < 0.01) between expression by TLR7^−/−^ DCs pretreated with ODN2088-Ctrl or ODN2088.

The TLR pathway has been traditionally associated with IFN-β production following pathogen recognition by the endosomal TLRs (TLR3, TLR7, and TLR9 in mice) (26). However, being considered a classical extracellular pathogen, recognition of *S. suis* has been mostly associated with surface TLRs (TLR1, TLR2, and TLR6) (13, 20). Consequently, the role of the TLR pathway in *S. suis*-induced IFN-β by DCs was evaluated. In the absence of the adaptor protein MyD88, used by the majority of TLRs, a significant decrease of IFN-β expression by DCs (*p* < 0.001) was observed with the three *S. suis* strains, corresponding to a near complete abrogation (Figure 3A). This result suggests that *S. suis*-induced IFN-β expression by DCs is overwhelmingly MyD88-dependent since only 5–10% of expression remained independent of MyD88 (Figure 3A).

Given the near complete MyD88-dependence of *S. suis-*induced IFN-β by DCs and the fact that the surface TLR2 (13, 20), and possibly TLR4 *via* the SLY (49), may be implicated in recognition of this pathogen, their role in *S. suis*-induced IFN-β by DCs was evaluated. TLR2 was not implicated in IFN-β expression by DCs following *S. suis* infection, regardless of the strain used (Figure 3B). Moreover, it was not possible to induce IFN-β expression following stimulation of wild-type DCs with PAM3CSK4 (for TLR1/2) and FSL-1 (for TLR2/6) (50), which are synthetic bacterial TLR2 ligands frequently used for most cell types (Figure S2 in Supplementary Material). However, TLR2 was involved in expression of the pro-inflammatory cytokines IL-6 and CXCL1 by DCs following infection with *S. suis* and stimulation by both TLR2 ligands (Figure S3 in Supplementary Material). These results demonstrate that though not capable of producing IFN-β following stimulation with control ligands (51), the cells remained responsive to TLR2-dependent *S. suis* stimulation. Surprisingly, TLR4 was partially implicated in IFN-β expression by DCs following infection with both the ST7 strain SC84 and the ST1 strain P1/7 (*p* < 0.05), which corresponded to a reduction of approximately 15%. TLR4 involvement was not observed with the ST25 strain 89-1591 (Figure 3B). A notable difference between the ST1/ST7 and ST25 strains is the absence of SLY in the latter strain. As such, IFN-β expression by wild-type and TLR4^−/−^ DCs was evaluated following infection with the SLY-deficient mutant (Figure 3C). Indeed, the wild-type and SLY-deficient strains induced similar levels of IFN-β by TLR4^−/−^ DCs, indicating that recognition of the SLY by TLR4 might contribute to the induction of this cytokine.

Despite the fact that *S. suis* has been described as remaining extracellularly, the largely surface TLR-independence of *S. suis*-induced IFN-β expression by DCs suggested that endosomal TLRs might participate in its induction. In accordance with *S. suis*-induced IFN-β production by DCs being mostly MyD88-dependent, the TLR3, which is MyD88-independent and recruits TRIF, was only minimally implicated, and only following infection with the SLY-negative ST25 strain 89-1591 (*p* < 0.05) (Figure 3D). Meanwhile, both TLR7 and TLR9 were responsible for IFN-β expression by DCs following *S. suis* recognition, regardless of the strain (Figure 3D). Indeed, their absence resulted in a 40–60% reduction of IFN-β expression, which was significantly lower when compared with expression by wild-type DCs (*p* < 0.01). In order to evaluate if recognition of *S. suis* by TLR7 and TLR9 was the result of a cooperative effect, DCs dually deficient for TLR7 and TLR9 were created by pretreating cells from either wild-type or TLR7^−/−^ mice with the TLR9 antagonist ODN2088 or its control, ODN2088-Ctrl (Figure 3E). Antagonizing wild-type DCs with ODN2088 resulted in a phenotype similar to that obtained using TLR9^−/−^ DCs. Use of ODN2088-Ctrl on wild-type and TLR7^−/−^ DCs confirmed the specificity of the treatment. When TLR7^−/−^ DCs were antagonized with ODN2088, creating a dual TLR7^−/−^/TLR9^−/−^ phenotype, a greater decrease, resulting in nearly 80% abrogation of IFN-β expression by wild-type DCs (*p* < 0.001), was observed regardless of the strain. Results of IFN-β by DCs following stimulation with the different purified or synthetic TLR ligands are presented in Figure S2 in Supplementary Material.

### The IRFs 1, 3, and 7 Play Important though Partially Redundant Roles in IFN-β Expression by DCs following *S. suis* Infection

**Figure 4:**
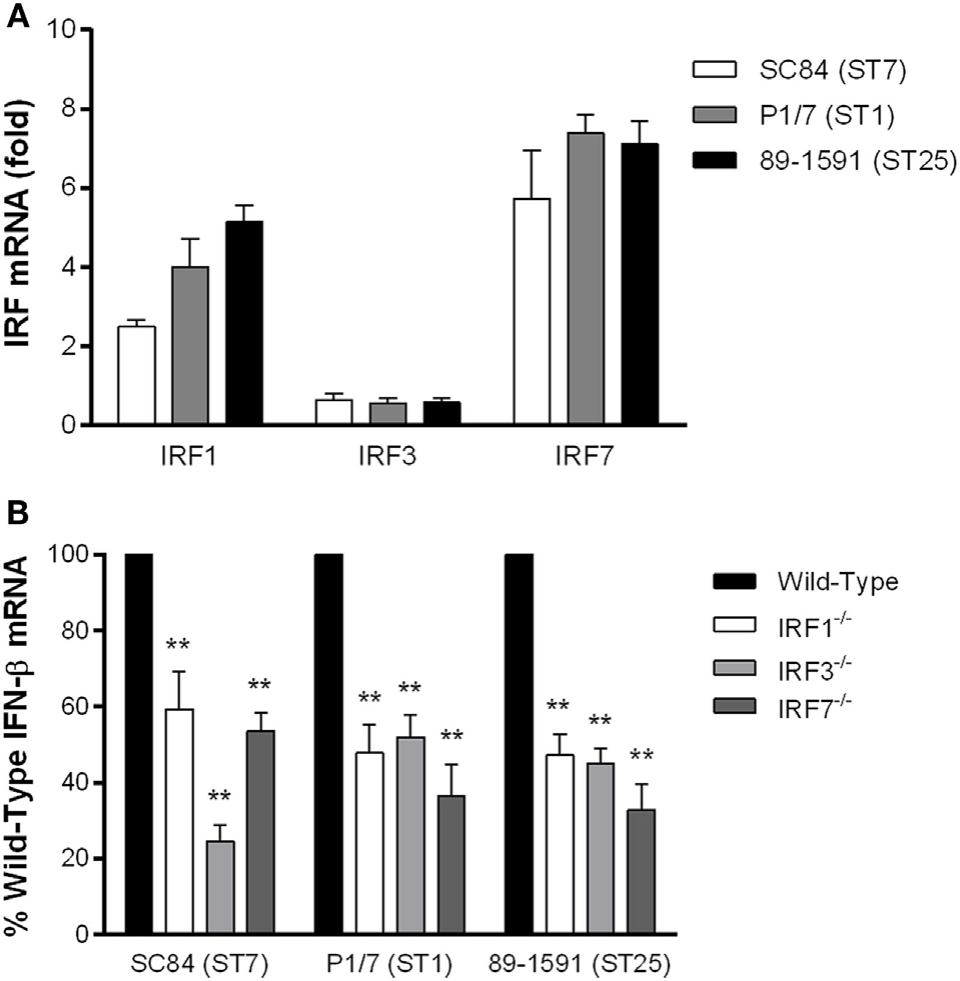
The interferon regulatory factors (IRFs) 1, 3, and 7 are involved in interferon (IFN)-β expression by dendritic cells (DCs) following infection with *Streptococcus suis*. *S. suis*-induced IRF1, IRF3, and IRF7 mRNA expression by DCs 6 h following infection with the different strains **(A)**. IFN-β mRNA expression induced by the different *S. suis* strains following infection of IRF1^−/−^, IRF3^−/−^, or IRF7^−/−^ DCs in comparison with cells from wild-type mice **(B)**. Data represent the mean ± SEM (*n* = 4). ** (*p* < 0.01) indicates a significant difference between expression by wild-type and deficient DCs.

Activation of the TLR pathway *via* the endosomal TLRs usually leads to phosphorylation of IRF3/IRF7 and subsequent production of IFN-β (26). Moreover, though IRF1 is typically associated with induction of type II IFN, crosstalk within the cell and/or simultaneous activation of various cellular pathways may result in IRF1 phosphorylation leading to IFN-β induction (52). Consequently, given that TLR7 and TLR9 are the main TLRs responsible for *S. suis*-induced IFN-β expression by DCs, the expression levels of IRF1, IRF3, and IRF7 in DCs were determined (Figure 4A). All three strains of *S. suis* induced upregulation of both IRF1 and IRF7, but not IRF3, with no significant differences among strains. However, IRF7 expression was significantly more upregulated than that of IRF1 (*p* < 0.05). Subsequently, in order to evaluate the role of IRF1, IRF3, and IRF7 in *S. suis*-induced IFN-β expression by DCs, cells isolated from wild-type and knock-out mice infected with the three strains of *S. suis* were evaluated. All three IRFs were significantly implicated in *S. suis*-induced IFN-β expression by DCs (*p* < 0.01), with reductions ranging between 40 and 70% (Figure 4B).

### *S. suis*-Induced IFN-β by DCs Requires Internalization and Phagosome Maturation

**Figure 5:**
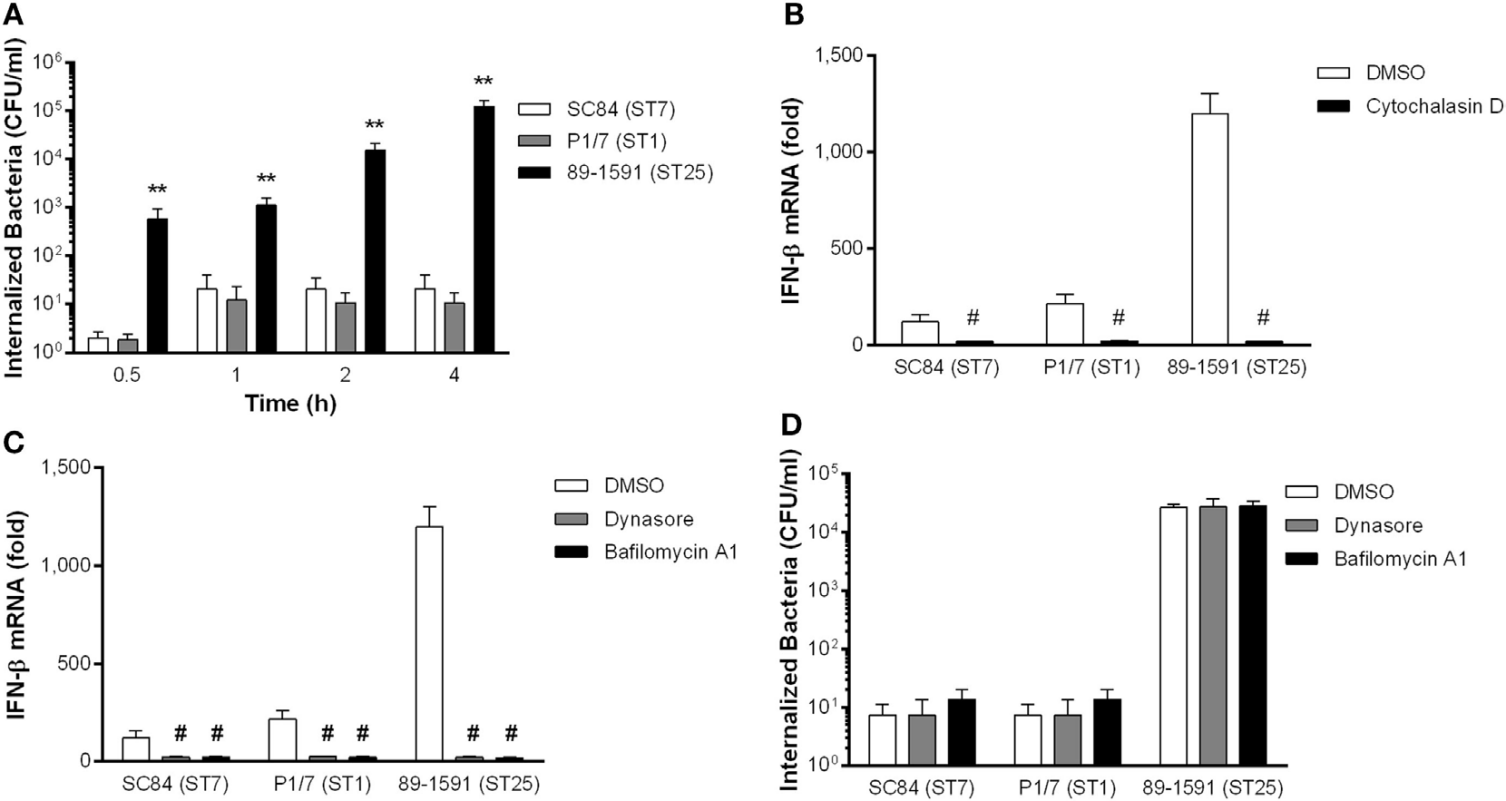
*Streptococcus suis*-induced interferon (IFN)-β expression by dendritic cells (DCs) requires internalization and phagosome maturation. Internalization kinetics of different *S. suis* strains by DCs **(A)**. Implication of actin polymerization (5 µM cytochalasin D) **(B)**, dynamin (8 µM dynasore), and vacuolar-type H^+^ ATPase-dependent phagosome acidification (1 µM bafilomycin A1) **(C)** on IFN-β mRNA expression by DCs 6 h following infection with *S. suis*. Effect of dynamin remodeling and phagosome acidification on internalization of *S. suis* by DCs 2 h following infection **(D)**. Data represent the mean ± SEM (*n* = 3). ** (*p* < 0.01) indicates a significant difference between 89-1591 and P1/7 or SC84; # (*p* < 0.001) between DCs treated with DMSO (vehicle) and DCs treated with the inhibitors (cytochalasin D, dynasore, or bafilomycin A1).

Previous studies with group A *Streptococcus* (GAS) and group B *Streptococcus* (GBS) have demonstrated that internalization of the pathogen and maturation of the phagosome are required for IFN-β production by DCs (28, 44). However, and differently from *S. suis*, these two pathogens are well-known to be internalized by phagocytes (53). Given that the endosomal TLR7 and TLR9 are implicated in *S. suis*-induced IFN-β expression by DCs, it was hypothesized that internalization could be a critical step, even for this classical extracellular pathogen whose CPS protects against phagocytosis (10–12, 54). However, no study has evaluated the capacity of DCs to internalize strains of *S. suis* other than ST1. Consequently, the kinetics of internalization of the three *S. suis* strains by DCs was evaluated. In accordance with previous studies (12), the well-encapsulated ST1 strain P1/7 was poorly internalized, even after 4 h of infection (Figure 5A). Similar results were obtained with the ST7 strain SC84, for which information was previously unavailable. On the other hand, the intermediate virulent ST25 strain 89-1591, which induces the highest levels of IFN-β, was surprisingly and significantly more internalized by DCs than the ST1 and ST7 strains (*p* < 0.01). Since the CPS is the most important antiphagocytic factor possessed by *S. suis* serotype 2, these results could have suggested that the strain 89-1591 was less encapsulated. However, it was possible to observe, using transmission electron microscopy, that the ST1 strain P1/7 (Figure S4A in Supplementary Material) and the ST25 strain 89-1591 (Figure S4B in Supplementary Material) are similarly well-encapsulated, suggesting that the greater internalization of strain 89-1591 by DCs is not the result of a thinner CPS.

In order to determine if *S. suis* internalization is indeed required for IFN-β expression by DCs, cells were pretreated with cytochalasin D, an inhibitor of actin polymerization, or its vehicle, DMSO. In the absence of actin polymerization, IFN-β expression was completely abrogated (*p* < 0.001) following infection with all three strains of *S. suis* (Figure 5B). In contrast to IFN-β expression, actin polymerization was only partially implicated in DC expression of IL-6 and CXCL1 following infection with the *S. suis* strain P1/7 (Figure S5 in Supplementary Material).

Once internalized by phagocytes, the pathogen will find itself within the phagosome, which must undergo maturation (55). Among the different proteins involved in internalization is the GTPase dynamin, which is required in the case of coated endosomal vesicles (56). As such, dynamin frequently contributes to endosomal signaling of IFN-β (28, 57). Indeed, when inhibiting dynamin activity using the inhibitor dynasore, a near complete abrogation of IFN-β expression (*p* < 0.001) was observed (Figure 5C). However, though dynamin was essential for *S. suis*-induced IFN-β expression by DCs, its role was not internalization-dependent since internalization levels of all three *S. suis* strains did not differ when inhibiting dynamin (Figure 5D).

Following phagosome formation, destruction of the pathogen requires acidification of the compartment, which occurs following fusion with the lysosome (58). This fusion, resulting in the creation of the phagolysosome, leads to acidification of the vesicle in which vacuolar-type H^+^ ATPases are implicated (59). Inhibition of these vacuolar-type H^+^ ATPases using bafilomycin A1 resulted in a significant (*p* < 0.001) and near complete abrogation of *S. suis*-induced IFN-β expression by DCs (Figure 5C). It should be noted that bafilomycin A1 treatment did not affect internalization of *S. suis* (Figure 5D).

### *S. suis* Nucleic Acids Are Responsible for Inducing IFN-β Expression by DCs following Endosomal Delivery

**Figure 6:**
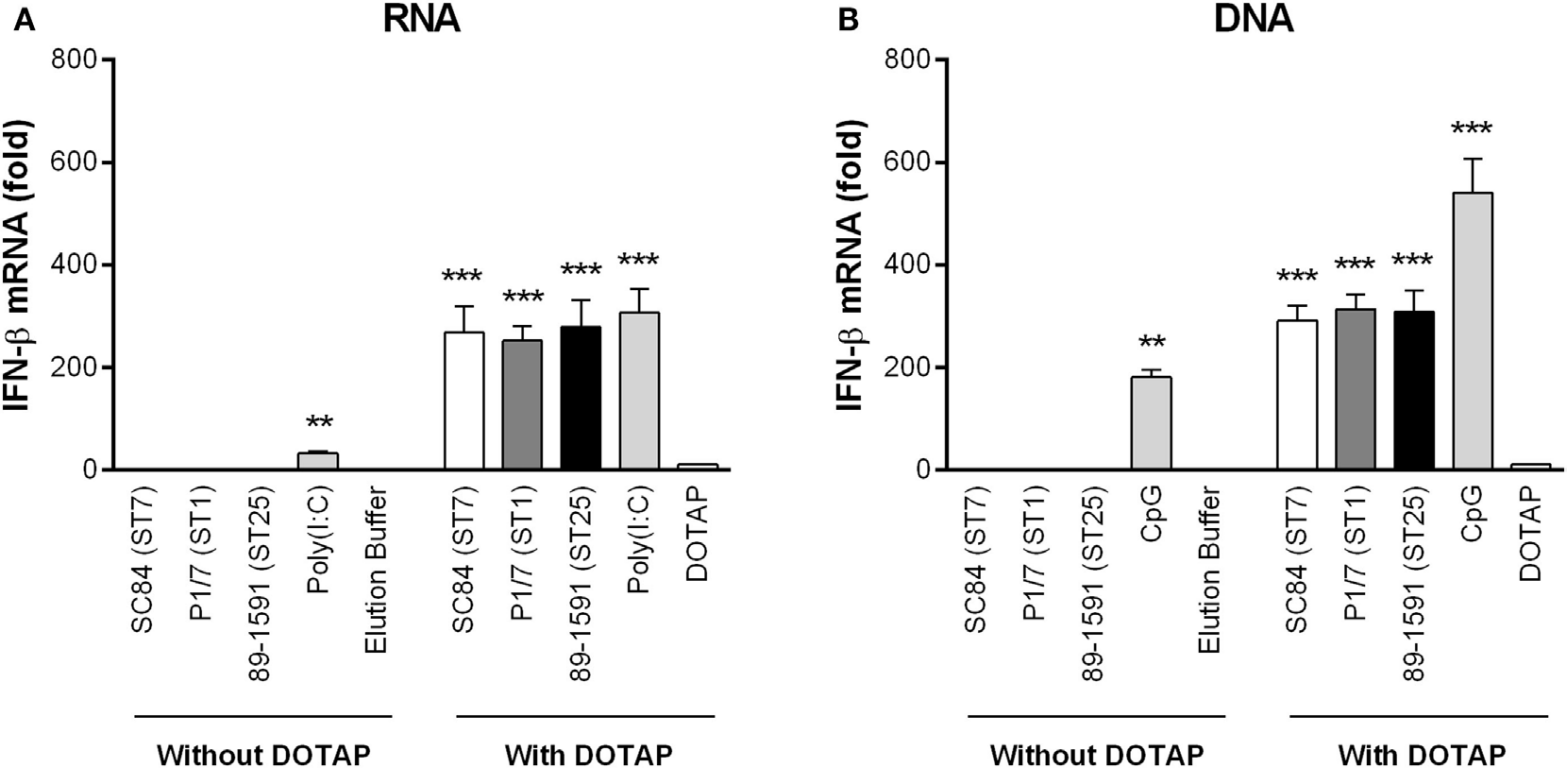
The *Streptococcus suis* nucleic acids are responsible for inducing interferon (IFN)-β expression by dendritic cells (DCs) following phagosomal delivery. IFN-β mRNA expression by DCs 6 h following transfection with RNA **(A)** or DNA **(B)** isolated from the different *S. suis* strains, poly(I:C) or CpG. Nucleic acids were complexed or not with DOTAP liposomal transfection agent prior to transfection of DCs. Data represent the mean ± SEM (*n* = 3). ** (*p* < 0.01) and *** (*p* < 0.001) indicate a significant difference with the elution buffer (negative control) or DOTAP alone (vehicle).

The requirement of internalization and phagosome maturation in *S. suis*-induced IFN-β expression by DCs suggests that destruction of the pathogen within the phagolysosome is necessary. TLR7 and TLR9 recognize single-stranded RNA and unmethylated CpG motifs of DNA, respectively (60, 61). To evaluate this hypothesis, bacterial RNA and DNA, isolated from all three *S. suis* strains, were complexed or not with DOTAP liposomal transfection agent which allows for delivery within the phagosome. As shown in Figure 6, both *S. suis* RNA (Figure 6A) and DNA (Figure 6B) induced significant levels of IFN-β in DCs (*p* < 0.001), but only when complexed with DOTAP. Similarly, the capacity of poly(I:C) (Figure 6A) and CpG (Figure 6B) to induce IFN-β by DCs was significantly increased when complexed with DOTAP (*p* < 0.001). Moreover, levels of IFN-β expression were similar between the different *S. suis* strains and between RNA and DNA.

### *S. suis*-Induced Type I IFN by DCs Modulates Autocrine Cytokine Production

**Figure 7:**
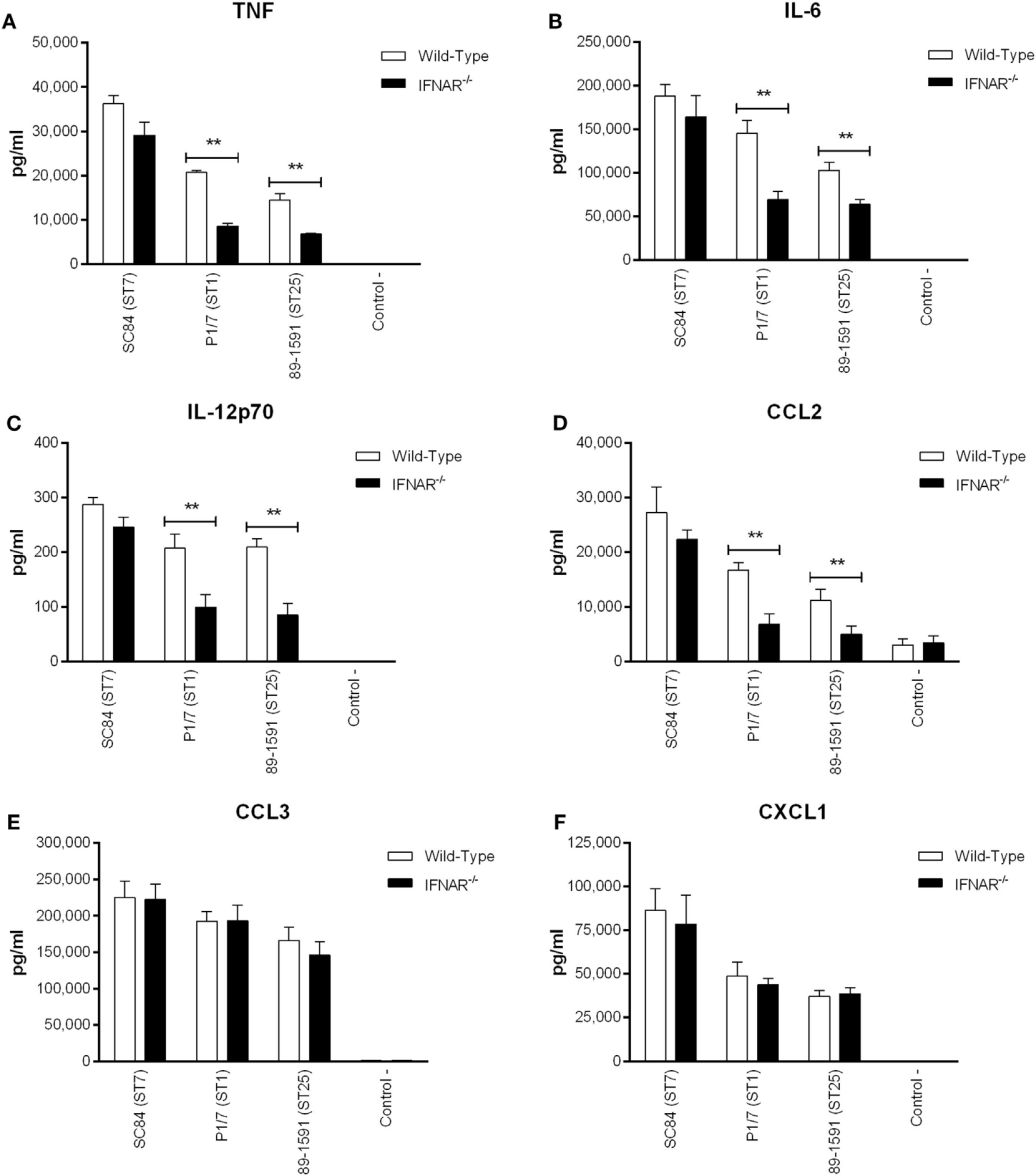
*Streptococcus suis*-induced type I interferon produced by dendritic cells (DCs) modulates autocrine cytokine production. Production of tumor necrosis factor **(A)**, interleukin (IL)-6 **(B)**, IL-12p70 **(C)**, CCL2 **(D)**, CCL3 **(E)**, and CXCL1 **(F)** by DCs 16 h following infection with the different *S. suis* strains. Data represent the mean ± SEM (*n* = 4). ** (*p* < 0.01) indicates a significant difference in cytokine production between wild-type and IFNAR^−/−^ DCs.

Once produced, type I IFN, including IFN-β, will bind to its receptor, IFNAR, located on the surface of most cell types, including DCs (26). Consequently, type I IFN can modulate autocrine production of other inflammatory mediators. As such, the production of certain inflammatory cytokines known to be induced by *S. suis* (20), by DCs derived from wild-type and IFNAR^−/−^ mice, was compared (Figure 7). A significant role of type I IFN (*p* < 0.01) was observed in TNF, IL-6, IL-12p70, and CCL2 (Figures 7A–D) production induced by the ST1 strain P1/7 and the ST25 strain 89-1591, but not by the ST7 strain SC84. On the other hand, no type I IFN-downstream modulation of CCL3 and CXCL1 production was observed for any of the strains tested.

### Type I IFN Is Beneficial for Host Survival during the *S. suis* Serotype 2 Systemic Infection: Implication in the Modulation of Systemic Inflammation Which Controls Blood Bacterial Burden

**Figure 8:**
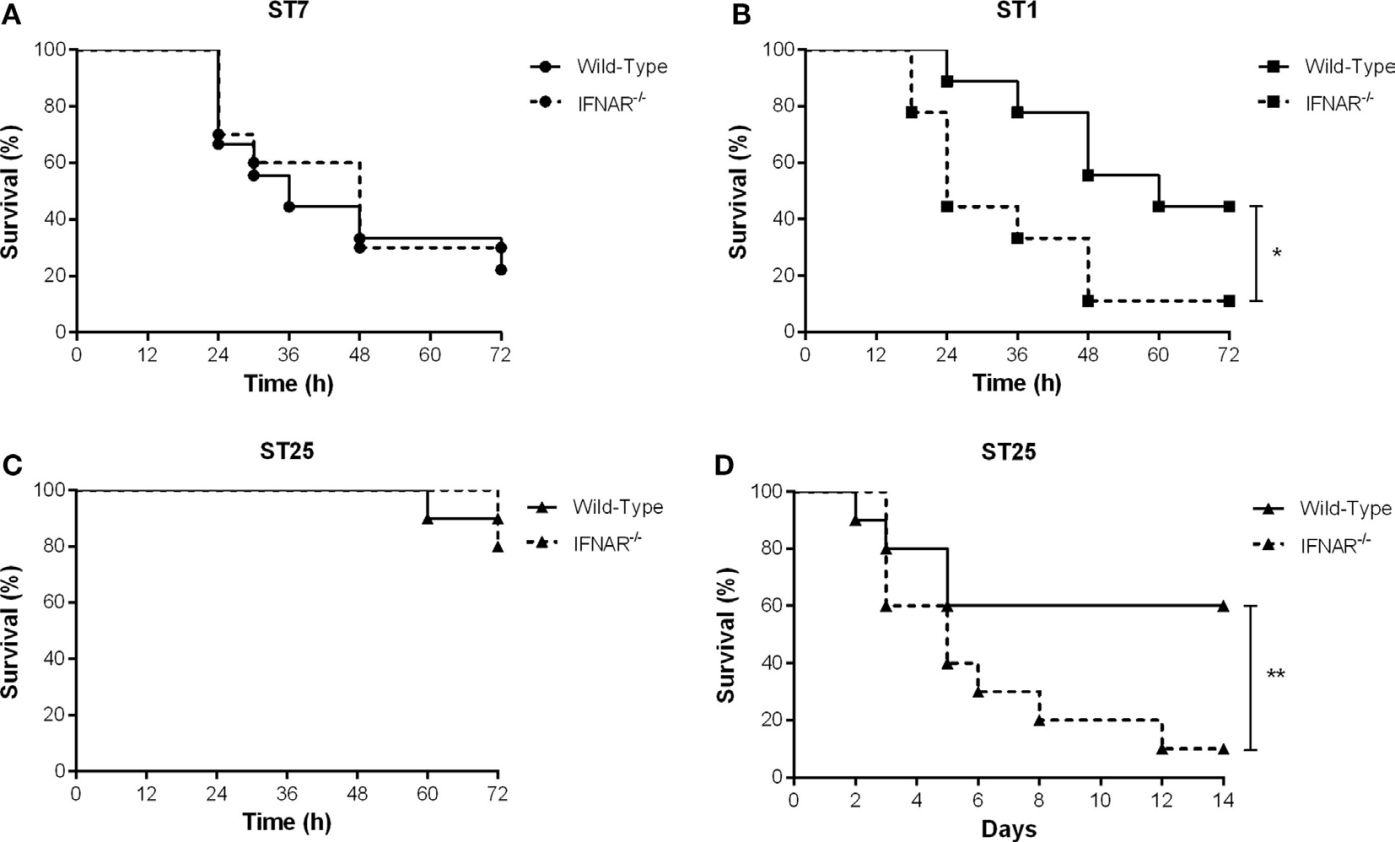
Type I interferon is beneficial for host survival following infection with intermediate virulent and virulent *Streptococcus suis* serotype 2 strains. Survival of wild-type and IFNAR^−/−^ mice infected with the different *S. suis* strains: the highly virulent ST7 strain SC84 **(A)**, the virulent ST1 strain P1/7 **(B)**, and the intermediate virulent ST25 strain 89-1591 **(C)** during the systemic infection [until 72 h post-infection (p.i.)]. Survival of wild-type and IFNAR^−/−^ mice infected with strain 89-1591 following both the systemic and central nervous system infections (14 days p.i.) **(D)**. Data represent survival curves (*n* = 15). * (*p* < 0.05) and ** (*p* < 0.01) indicate a significant difference between survival of wild-type and IFNAR^−/−^ mice.

The *in vitro* production of IFN-β by DCs observed in this study, coupled with the upregulation of this cytokine in mice infected with *S. suis* (3), suggests that IFN-β may be implicated in the balance and/or exacerbation of the systemic inflammation induced by this pathogen, and subsequently, host survival. Consequently, the role of type I IFN during the *S. suis* systemic infection caused by the three strains was evaluated using a well-standardized intraperitoneal C57BL/6 mouse model of infection comparing wild-type and IFNAR^−/−^ mice (45). No role of type I IFN was observed in mouse survival during the systemic infection with the highly virulent ST7 strain SC84, with wild-type or IFNAR^−/−^ mice equally succumbing to the infection (Figure 8A). Meanwhile, type I IFN played a significant role in the survival of mice infected with the virulent ST1 strain P1/7 (*p* < 0.05), with IFNAR^−/−^ mice succumbing at a greater rate than their wild-type counterparts (45% of wild-type mice survived the systemic infection vs. only 10% of the IFNAR^−/−^ mice) (Figure 8B). Interestingly, no role of type I IFN was observed during early systemic infection (first 72 h p.i.) with the intermediate virulent ST25 strain 89-1591 (Figure 8C), which induced high levels of IFN-β *in vitro*. However, given the lower virulence of this strain, which caused only 10–20% of mortality at 72 h p.i., this result was not entirely surprising. Less virulent *S. suis* strains are known to cause a delayed mortality, usually by meningitis, due to persistent bacteremia (4). As such, survival of mice was evaluated until 14 days p.i. (Figure 8D). Four days p.i., mortality in the wild-type group increased but then remained stable until the end of the experiment. On the other hand, IFNAR^−/−^ mice were significantly more susceptible to the infection than their wild-type counterparts (*p* < 0.01) (Figure 8D). Taken together, these results suggest that type I IFN plays a beneficial role during the *S. suis* infection, at least for the ST25 and, to a certain extent, the ST1 strains, but not with the ST7 strain.

**Figure 9:**
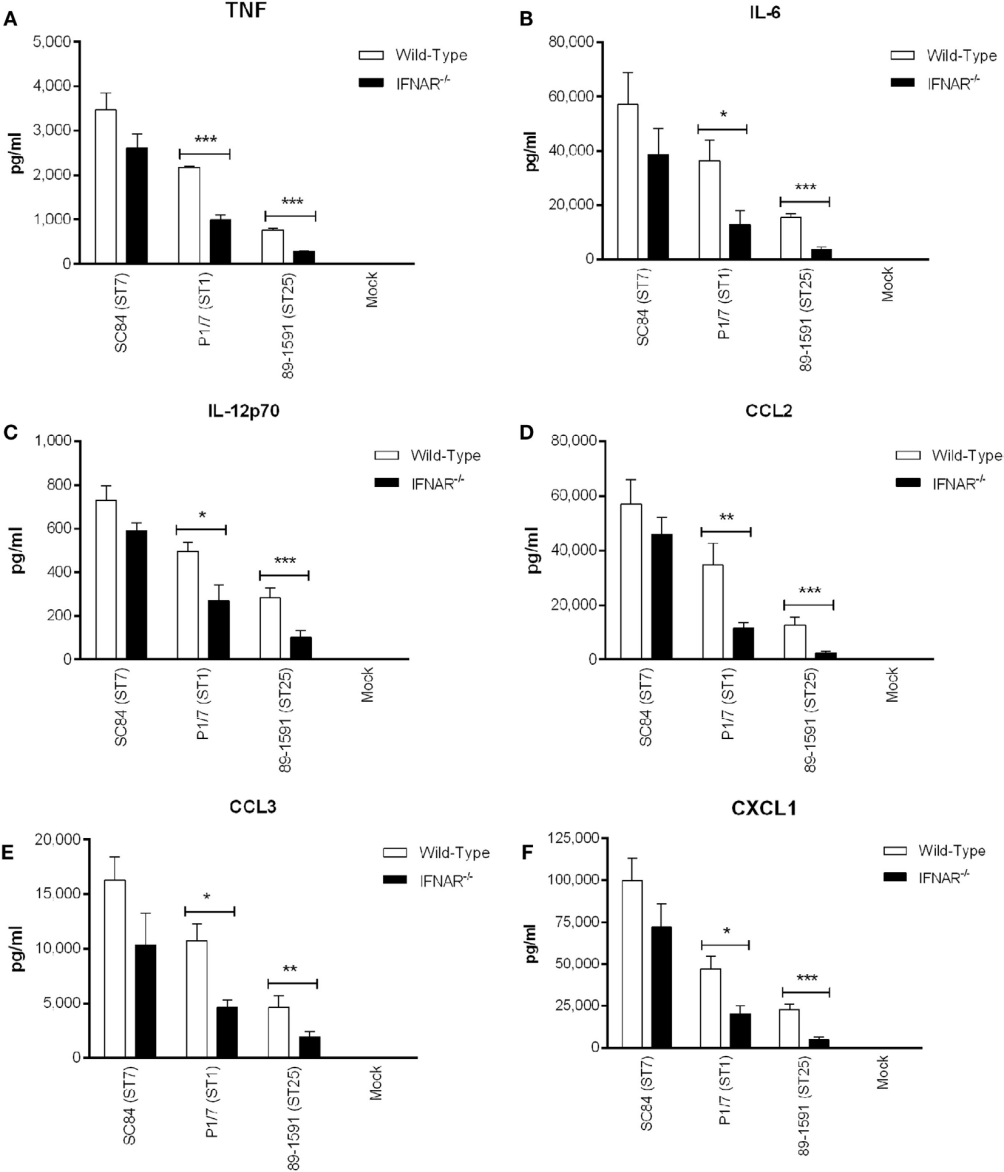
Type I interferon modulates plasma pro-inflammatory cytokines involved in *Streptococcus suis*-induced systemic inflammation. Plasma levels of tumor necrosis factor **(A)**, interleukin (IL)-6 **(B)**, IL-12p70 **(C)**, CCL2 **(D)**, CCL3 **(E)**, and CXCL1 **(F)** in wild-type and IFNAR^−/−^ mice 12 h following infection with the different *S. suis* strains. Data represent the mean ± SEM (*n* = 8). * (*p* < 0.05), ** (*p* < 0.01), and *** (*p* < 0.001) indicate a significant difference in plasma levels between wild-type and IFNAR^−/−^ mice.

Host death during the *S. suis* systemic infection is usually the result of uncontrolled blood bacterial burden resulting from excessive bacterial growth, concomitant with an exacerbated systemic inflammatory response (4, 62). As such, the role of type I IFN in aggravated inflammation was evaluated by measuring plasma (systemic) cytokines of both wild-type and IFNAR^−/−^ mice 12 h p.i., as previously described (Figure 9) (4, 45). For TNF, IL-6, IL-12p70, CCL2, CCL3, and CXCL1 (Figures 9A–F), a significant decrease in the levels produced by IFNAR^−/−^ mice, in comparison to their wild-type counterparts, was only observed for the strains P1/7 and 89-1591 (*p* < 0.05), with the difference being more pronounced in mice infected with the strain 89-1591.

**Figure 10:**
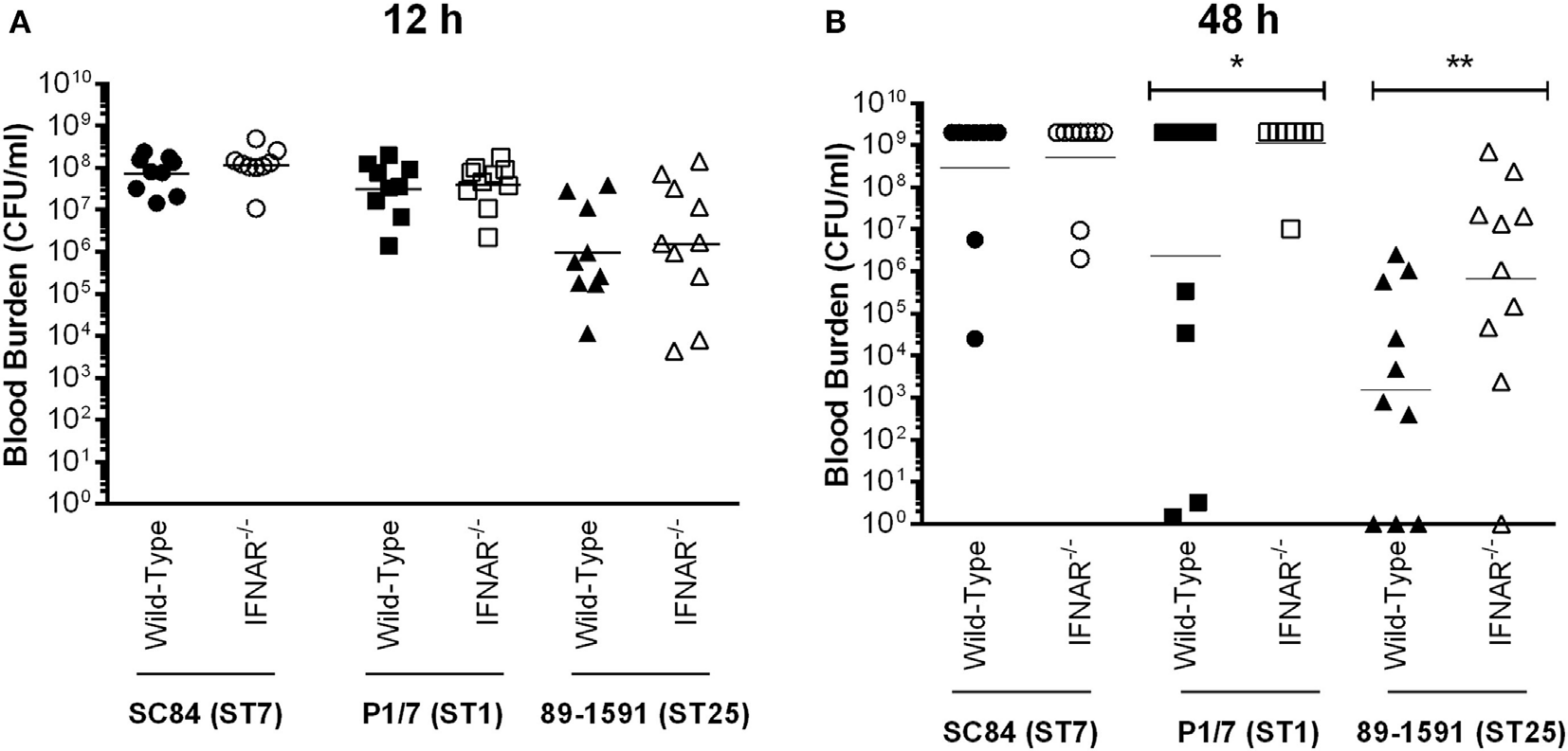
Type I interferon is required for control of blood bacterial burden following infection with intermediate virulent and virulent *Streptococcus suis* serotype 2 strains. Blood bacterial burden of wild-type and IFNAR^−/−^ mice infected with the different *S. suis* strains 12 h post-infection (p.i.) **(A)** or 48 h p.i. **(B)**. Data represent the geometric mean (*n* = 15). A blood bacterial burden of 2 × 10^9^ CFU/mL, corresponding to the average burden upon euthanasia, was attributed to euthanized mice. * (*p* < 0.05) and ** (*p* < 0.01) indicate a significant difference between blood bacterial burden of wild-type and IFNAR^−/−^ mice.

The second factor responsible for host death during the *S. suis* systemic infection is uncontrolled blood bacterial burden (4). No differences were observed between the acute blood bacterial burden of wild-type and IFNAR^−/−^ mice early after infection (12 h), regardless of the strain (Figure 10A). However, since differences in mortality only became important at later time points, the effect of type I IFN during the *S. suis* infection was possibly not immediate, but rather delayed. Indeed, blood bacterial burden of mice infected with the ST1 strain P1/7 and the ST25 strain 89-1591, but not of those infected with the ST7 strain SC84, was significantly higher in IFNAR^−/−^ mice 48 h p.i. than in their wild-type counterparts (*p* < 0.05) (Figure 10B). Consequently, type I IFN is implicated in the modulation of systemic inflammatory mediators required for control of blood bacterial burden.

## Discussion

*Streptococcus suis* serotype 2, an important porcine and emerging human pathogen, has always been considered a prototypical extracellular bacterium whose CPS confers important anti-phagocytic properties (6, 18). Consequently, recognition of *S. suis* by the innate immune response was long thought to occur following interaction of bacterial lipoproteins (or other cell wall components) with surface-associated receptors, in particular TLR2 (13, 20, 21, 63). More recently, however, partial implication of the endosomal TLR9 and cytoplasmic nucleotide-binding oligomerization domain-containing protein (NOD) 2 in recognition of *S. suis* by DCs was reported (20), suggesting that *S. suis* or its products might also activate cellular intracellular pathways (in-out signals). Nonetheless, the implications of such intracellular receptors in the pathogenesis of the infection caused by this bacterial pathogen were, so far, unknown.

Since *S. suis* was previously reported to upregulate levels of IFN-β *in vivo* (3), but not IFN-α, the ability of DCs and macrophages, which are usually important sources of pro-inflammatory mediators, to produce IFN-β following *S. suis* infection was evaluated. Meanwhile, information regarding type III IFN is limited to a single *in vitro* study (24). In response to *S. suis*, DCs produced higher levels of IFN-β than macrophages, suggesting different activation levels and/or intrinsic differences in cytokine production by both cell types, resulting in part from the activation of varying signaling pathways and cascades (44). Since results obtained with positive controls revealed that DCs and macrophages are both able to induce high IFN-β levels, the differential activation of these two cell types by *S. suis* reflects an intrinsic feature of this pathogen, a characteristic that can be extended to other pathogenic streptococci (28, 44).

Lachance et al. suggested that *in vivo* levels of IFN-β were inversely associated with virulence of the strain used (3), a fact that was confirmed in this study with DCs, where the intermediate virulent ST25 strain 89-1591 induced higher levels of IFN-β than the virulent ST1 and ST7 strains. This inverse association is not a trait unique to the strain used, since two other intermediate virulent ST25 strains, as well as an additional ST1 strain (31533), presented results similar to their respective prototypical ST strains. To our knowledge, the inversed association of virulence with IFN-β induction has not been previously described for other bacterial pathogens. However, this association does not apply to strains of highly virulent to virulent phenotypes, as ST7 and ST1 strains induce similar levels of IFN-β.

The TLR pathway has been traditionally associated with IFN-β induction following recognition by the endosomal TLRs (26), yet, as aforementioned, recognition of *S. suis* has been mostly demonstrated to occur *via* surface TLRs (13, 20). *S. suis-*induced IFN-β by DCs was MyD88-dependent, indicating that the TLR pathway is almost exclusively implicated in its production. However, even though production of many *S. suis*-induced pro-inflammatory cytokines by DCs has been reported to be mainly TLR2-dependent (20), we were unable to induce TLR2-dependent IFN-β expression by DCs. Though TLR2 activation can result in IFN-β production by macrophages, it was previously suggested that this is not the case for DCs (64, 65). On the other hand, a partial role of TLR4 was observed in IFN-β expression induced by ST1/ST7 strains, but not by ST25 strains: this activation may be related to SLY production. Indeed, this toxin was previously reported to be recognized by TLR4 expressed on peritoneal macrophages (49).

The limited contribution of surface TLRs to *S. suis*-induced IFN-β expression by DCs suggested that endosomal TLRs, of which TLR7 and TLR9 are MyD88-dependent (61), might participate in its induction. The involvement of endosomal TLRs in recognition of *S. suis* has been little evaluated since it has been considered an extracellular pathogen. Consequently, it was unexpected that the TLR7 and TLR9 were equally and primarily responsible for *S. suis*-induced IFN-β. Though TLR7 and to a lesser extent TLR9 were responsible for IFN-β production following recognition of GBS (44), no individual TLR could be identified for GAS (28). Even though *S. suis* and GBS share a similarity in this regard, the pathogenesis of these two encapsulated bacteria greatly differs: the most important difference between these two pathogens is that while *S. suis* is a classical extracellular bacterium protected from phagocytosis by its CPS, well-encapsulated GBS is highly internalized (53). In the case of GBS, IFN-β production by DCs was shown to be completely dependent on IRF1 and only partially IRF7-dependent (44). Interestingly, IRF1, IRF3, and IRF7 were all implicated in *S. suis*-induced IFN-β by DCs, suggesting a partial redundancy in signaling pathways, not observed for GBS. Indeed, localization of pathogens within the phagosome usually triggers IRF1 and IRF7 (44). Furthermore, IFN-β induced by TLR9 agonists results in IRF1 activation *via* a phagosome-dependent pathway (66). Participation of IRF3 may be the result of TLR3 or TLR4 activation by SLY-negative and SLY-positive strains, respectively, *via* a MyD88-independent, TRIF-dependent pathway (52). While IRF3 was partially implicated in *S. suis*-induced IFN-β by DCs, its expression was not modulated following infection with *S. suis*, unlike that of IRF1 and IRF7. This suggests that other processing steps could be important for regulation of IRF3. Indeed, it was previously reported that unlike IRF1 and IRF7, expression of IRF3 is constitutive, with phosphorylation, rather than transcription modulating its activation (41). Moreover, feedback loops resulting from crosstalk between pathways could also be responsible for implication of IRF3 (41). In agreement with activation of these intracellular pathways, *S. suis*-induced IFN-β levels inversely correlate with strain-dependent capacity to resist phagocytosis. This hypothesis is supported by the significantly higher levels of IFN-β observed in this study when using the non-encapsulated ST1 mutant strain, which was previously demonstrated to be highly internalized by DCs (20), and by the complete abrogation of IFN-β induction following blockage of actin-dependent internalization. Interestingly, non-encapsulated *S. suis* strains have traditionally been shown to increase cytokine induction by hindering recognition of surface cell wall components, responsible for cell activation by surface-associated receptors (12, 13, 20), mechanism that would not be involved in IFN-β modulation.

Previous studies with GAS and GBS have demonstrated that internalization of the pathogen and maturation of the phagosome are required for IFN-β production by DCs (28, 44). Results obtained in this study demonstrate that though dynamin is required for IFN-β production, this protein is implicated in early pre-acidification steps of phagosome maturation rather than in phagosome formation as evidenced by lack of effect on *S. suis* internalization. Subsequent acidification of the phagosome is required for IFN-β expression by DCs following infection with *S. suis*, suggesting that bacterial processing *via* hydrolytic degradation is essential for the liberation of TLR7 and TLR9 ligands (28, 44). These results indicate that bacterial nucleic acids were the ligands of TLR7 and TLR9. Indeed, both bacterial RNA, and to a lesser extent, DNA, from GAS and GBS are also responsible for IFN-β production by DCs following stimulation of TLR7 and TLR9 (28, 44). In contrast to GAS and GBS, however, the *S. suis* RNA and DNA induced similar levels of IFN-β, suggesting that both nucleic acids have comparable stimulatory effects. This is supported by the dual implication of TLR7 and TLR9 in *S. suis*-induced IFN-β production by DCs. Nucleic acids isolated from the three *S. suis* strains induced similar levels of IFN-β by DCs, indicating that regardless of virulence, the different *S. suis* strains possess similar stimulatory properties and differences observed would be mainly attributed to intracellular bacterial levels. The importance of nucleic acid localization within the endosome following internalization and pathogen degradation as critical steps of IFN-β induction by *S. suis* is further evidenced by the complete lack of IFN-β expression when the nucleic acids were not complexed with DOTAP. In agreement, while the TLR agonists poly(I:C) and CpG induced IFN-β in the absence of DOTAP, being powerful activators, their potency was significantly increased when complexed with DOTAP.

Once produced, type I IFN will bind to its receptor, IFNAR, located on the surface of most cell types, including DCs (26). This autocrine effect modulates *S. suis*-induced pro-inflammatory cytokines by DCs, as well as distal production of cytokines and chemokines by other cells as observed *in vivo* during *S. suis* infection. The IFNAR downstream effect is complex as evidenced by a lack of effect on DC chemokine production at the single-cell level (67). Yet, when analyzing the global systemic response, the release of the chemokines CCL3 and CXCL1 is indeed modulated by the type I IFN pathway. This pathway was previously demonstrated to amplify TNF signaling following infection with GBS, *S. pneumoniae*, and *E. coli* (29). Pro-inflammatory cytokine signaling is the result of a cascade triggered by TNF, among other mediators, leading to production of IL-6 and IL-12p70 (68). An amplification of these downstream cytokines by type I IFN is thus expected. Similarly, type I IFN was shown to modulate the recruitment of myeloid cells by influencing CCL2 signaling during infection with the intracellular pathogens *Listeria monocytogenes* and *Mycobacterium tuberculosis* (69, 70). Given that IFNAR downstream signaling is complex, it could be interesting to evaluate the role of the suppressor of cytokine signaling (SOCS) protein family since they are important regulators of the JAK-STAT pathway associated with IFNAR (71). Indeed, previous studies have demonstrated that *S. suis* induces upregulation of SOCS3 in mice and pigs following infection, with SOCS3 having been reported to block the catalytic site of JAK, thus inhibiting phosphorylation of STAT and suppressing IFNAR-induced effects (3, 71, 72). Taken together, these results indicate a mechanism complementary to surface-associated receptor activation whereby higher internalization of *S. suis* leads to increased IFN-β induction and subsequent regulation of the pro-inflammatory loop. Our data also suggest that this IFN-β-modulated inflammatory response contributes to control bacterial burden during *S. suis* infection and improves the clinical outcome of infected animals.

It has been previously reported with other pathogens that the induction of IFN-β may be either beneficial or detrimental for the host, as shown using experimental infections. For example, a similar beneficial role was described for GAS (28), GBS (29, 44), and *S. pneumoniae* (29–31). On the other hand, the induction of a strong type I IFN response may also be considered a key factor in early progression of invasive *S. pneumoniae* beyond the lung during development of invasive pneumococcal disease (32). Moreover, type I IFN is associated with suppression of the innate response to certain bacterial infections, such as *L. monocytogenes* and *Francisella tularensis*, resulting in hindered bacterial clearance and deleterious host effects (52).

Taken together, type I IFN is produced by the host following *S. suis* infection and contributes to a regulated inflammatory response. In the case of the intermediate virulent ST25 strain, the elevated IFN-β production modulates systemic pro-inflammatory mediators and appears responsible for the decreased blood bacterial burdens, which ultimately results in a reduction of meningitis and increased host survival. Indeed, it was previously reported that persistent blood bacterial burden is a prerequisite for the development of *S. suis* meningitis (4). Albeit lower levels of IFN-β production, a beneficial effect is also noticed after infection with the virulent ST1 strain. In contrast, type I IFN is unable to counteract the exacerbated inflammatory response and/or bacterial burden induced by the highly virulent ST7 strain. This observation might be related to its genetic particularities, including the presence of a pathogenicity island, and its capacity to induce exaggerated inflammation unparalleled by other *S. suis* strains, resulting in streptococcal toxic shock-like syndrome characterized by a cytokine storm (73–75).

In conclusion, this study demonstrates that, depending on the virulence level of the strain, type I IFN is involved in host defense during the *S. suis* infection by participating in clearance of blood bacterial burden and/or modulation of systemic inflammation. Results also showed that the lower virulence of the North American serotype 2 ST25 strains might be related to a lower resistance to phagocytosis that would lead to increased intracellular receptor activation with consequent IFN-β induction. Underlying mechanisms involved in the control of inflammation and subsequent bacterial burden could help to develop control measures for this important zoonotic infection.

## Ethics Statement

This study was carried out in accordance with the recommendations of the guidelines and policies of the Canadian Council on Animal Care and the principles set forth in the Guide for the Care and Use of Laboratory Animals. The protocols and procedures were approved by the Animal Welfare Committee of the University of Montreal (protocol number rech-1570).

## Author Contributions

Conceived and designed the experiments: J-PA, MS, and MG. Performed the experiments: J-PA, AS, and DR. Analyzed the data: J-PA, MS, and MG. Provided research tools: KM and JX. Contributed to the writing of the manuscript: J-PA, MS, and MG. All the authors have read and approved the manuscript.

## Conflict of Interest Statement

The authors declare that the research was conducted in the absence of any commercial or financial relationships that could be construed as a potential conflict of interest.

## Abbreviations

CCL, C–C motif chemokine ligand; CFU, colony-forming unit; CPS, capsular polysaccharide; CXCL, C-X-C motif chemokine ligand; DC, dendritic cell; GAS, group A *Streptococcus*; GBS, group B *Streptococcus*; IFN, interferon; IFNAR, interferon-α/β receptor; IL, interleukin; IRF, interferon regulatory factor; JAK, Janus kinase; p.i., post-infection; SLY, suilysin; SOCS, suppressor of cytokine signaling; ST, sequence type; STAT, signal transducer and activator of transcription; THB, Todd Hewitt broth; TLR, Toll-like receptor; TNF, tumor necrosis factor.
