# Supplementary material for: Type I Interferon Induced by Streptococcus suis Serotype 2 is Strain-Dependent and May Be Beneficial for Host Survival
Source: Front Immunol. 2017 Aug 28;8:1039. doi: 10.3389/fimmu.2017.01039 (PMC5581389; doi:10.3389/fimmu.2017.01039)
Supplement: Supplementary file 1 [file data_sheet_1.pdf]

Supplementary Material

Type I Interferon Induced by *Streptococcus suis* Serotype 2 is Strain-Dependent and May Be Beneficial for Host Survival

Jean-Philippe Auger, Agustina Santinon, David Roy, Karen Mossman, Jianguo Xu, Mariela Segura, and Marcelo Gottschalk*

* **Correspondence:** Marcelo Gottschalk; marcelo.gottschalk@umontreal.ca

Supplementary Table

Table S1. Primer sequences used for real-time qPCR

Supplementary Figures

Figure S1. Intermediate virulent *Streptococcus suis* ST25 strains induce high levels of IFN-β expression by dendritic cells
Figure S2. Ligands of the different Toll-like receptors evaluated in this study, with the exception of TLR2, induce IFN-β by dendritic cells
Figure S3. IL-6 and CXCL1 induced by *Streptococcus suis* and bacterial TLR2 ligands partially require TLR2 expression by dendritic cells
Figure S4. The intermediate virulent *Streptococcus suis* strain 89-1591 is highly encapsulated
Figure S5. *Streptococcus suis*-induced IL-6 and CXCL1 expression by dendritic cells is partially internalization-dependent

Table S1. Primer sequences used for real-time qPCR.

Gene	Forward (F) and reverse (R) primers
Atp5b	F: ACC AGC CCA CCC TAG CCA CC<br>R: TGC AGG GGC AGG GTC AGT CA
Gapdh	F: CCC GTA GAC AAA ATG GTG AAG<br>R: GAC TGT GCC GTT GAA TTT G
Ifnb	F: CCC AGT GCT GGA GCC ATT GT<br>R: CCC TAT GGA GAT GAC GGA GA
Irf1	F: AGG CAT CCT TGT TGA TGT CC<br>R: AAT TCC AAC CAA ATC CCA GG
Irf3	F:GAT GGC TGA CTT TGG CAT CT<br>R: ACC GGA AAT TCC TCT TCC AG
Irf7	F: AGC ATT GCT GAG GCT CAC TT<br>R: TGA TCC GCA TAA GGT GTA CG
Il6	F: ATG GTA GCT ACC AAA CTG GAT<br>R: TGA AGG ACT CTG GCT TTG TCT
Cxcl1	F: TCT CCG TTA CTT GGG GAC AC<br>R: CCA CAC TCA AGA ATG GTC GC

Auger *et al.*

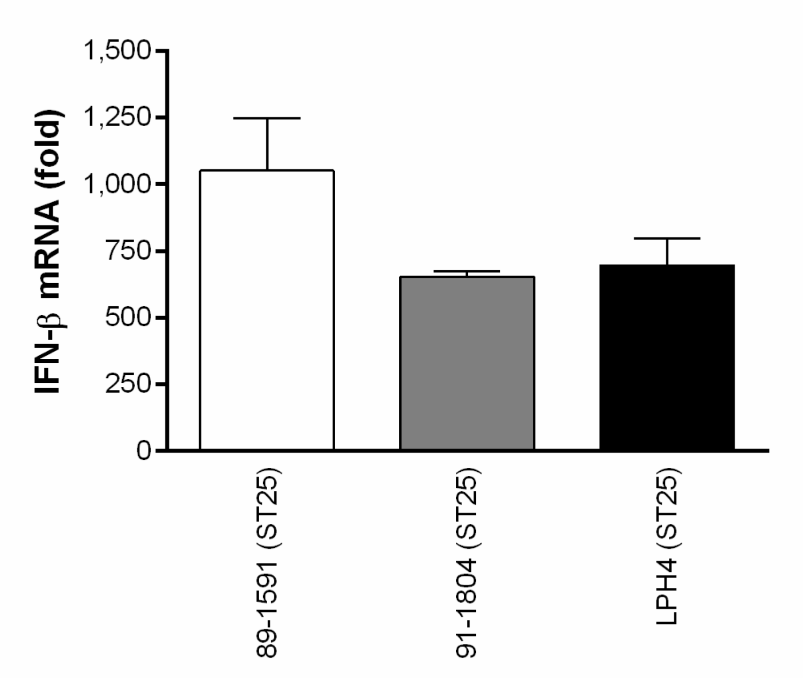

Figure S1. Intermediate virulent *Streptococcus suis* ST25 strains induce high levels of IFN-β expression by dendritic cells. IFN-β mRNA expression by dendritic cells 6 h following infection with the intermediate virulent ST25 strains 89-1591, 91-1804, and LPH4. Virulence of strains 91-1804 and LPH4 was previously described. Data represent the mean ± SEM (n = 4).

Auger *et al.*

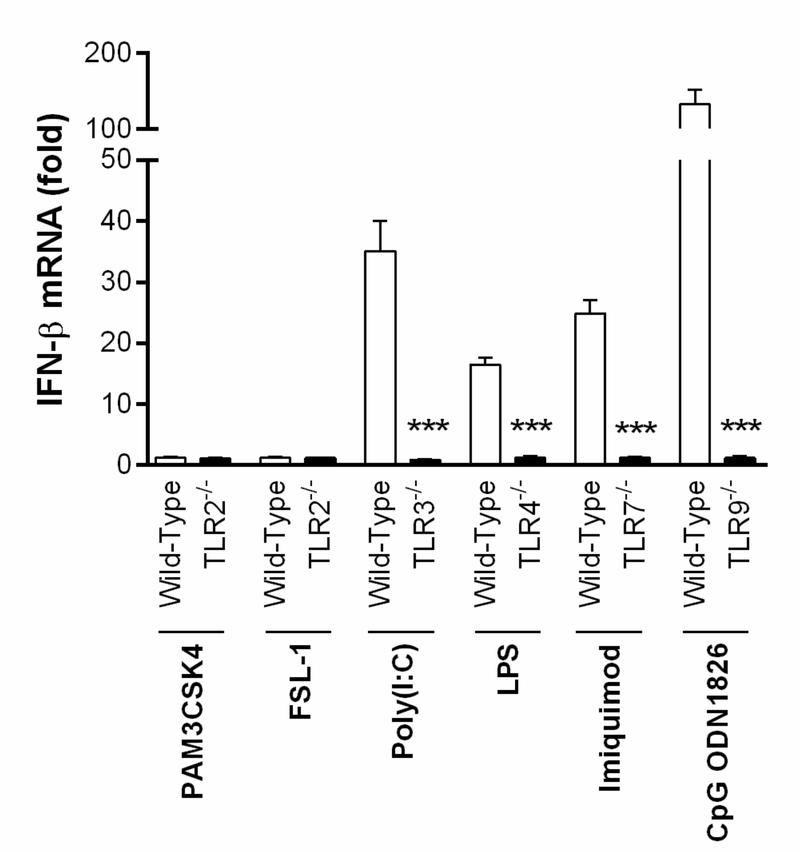

Figure S2. Ligands of the different Toll-like receptors (TLRs) evaluated in this study, with the exception of TLR2, induce IFN-β by dendritic cells. IFN-β mRNA expression by wild-type and deficient dendritic cells 6 h following activation with the different TLR ligands: 1 µg/mL PAM3CSK4 (TLR1/2), 1 µg/mL FSL-1 (TLR2/6), 10 µg/mL poly(I:C) (TLR3), 100 ng/mL lipopolysaccharide (LPS) (TLR4), 5 µg/mL imiquimod (TLR7), and 1 µM CpG ODN1826 (TLR9). Data represent the mean ± SEM (n = 4). *** ($p < 0.001$) indicates a significant difference in IFN-β expression by wild-type and deficient dendritic cells.

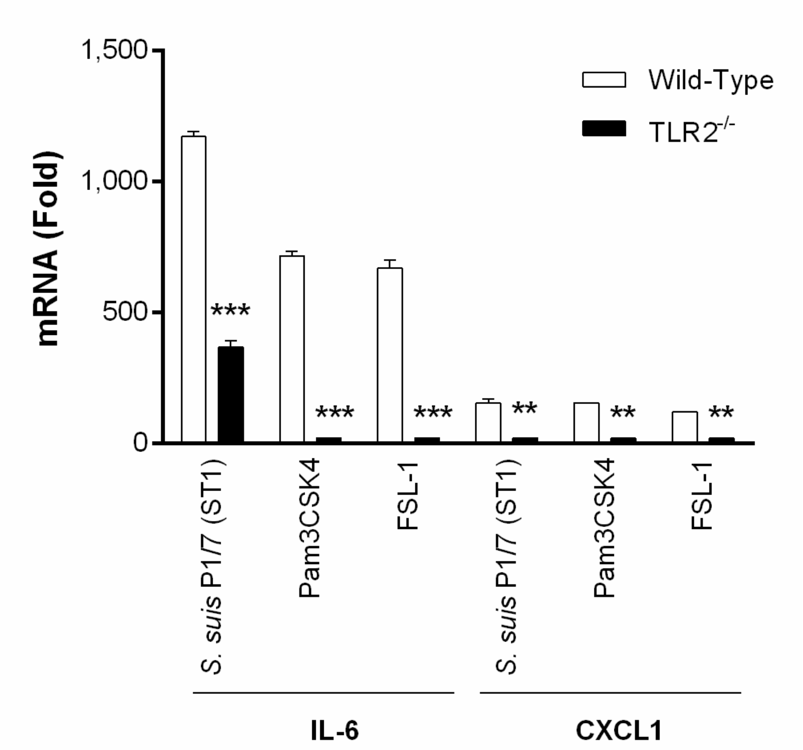

Figure S3. IL-6 and CXCL1 induced by *Streptococcus suis* and bacterial TLR2 ligands partially require TLR2 expression by dendritic cells. IL-6 and CXCL1 expression by wild-type or TLR2$^{-/-}$ dendritic cells 6 h following infection with *S. suis* ST1 strain P1/7 or activation with the TLR2 ligands PAM3CSK4 (1 µg/mL) and FSL-1 (1 µg/mL). Data represent the mean ± SEM (n = 3). ** ($p < 0.01$) and *** ($p < 0.001$) indicate a significant difference between expression by wild-type and TLR2$^{-/-}$ dendritic cells.

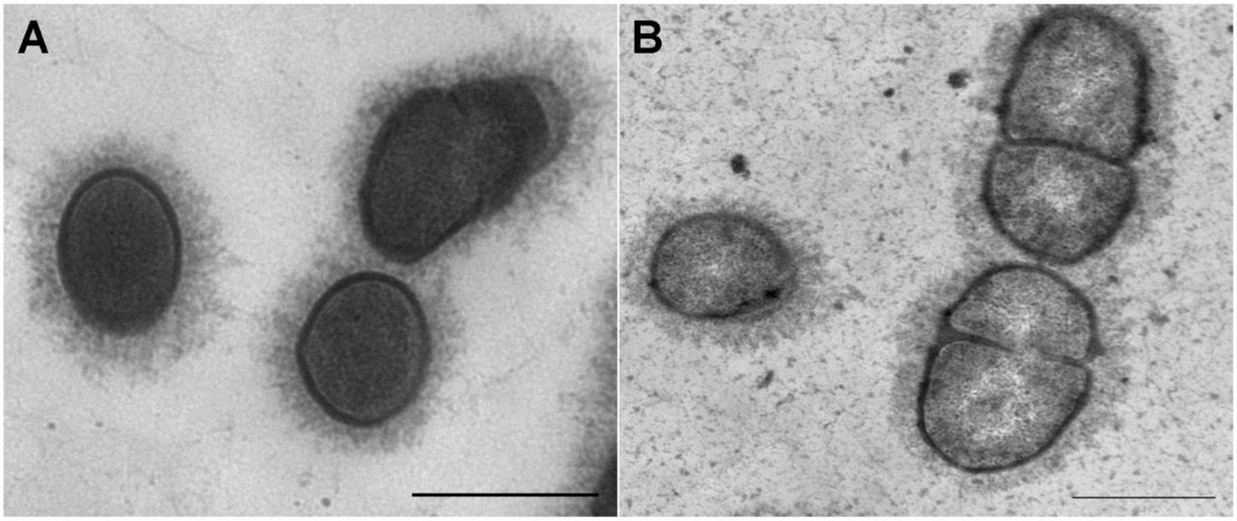

Figure S4. The intermediate virulent *Streptococcus suis* strain 89-1591 is highly encapsulated. Transmission electron micrographs following antibody stabilization of the capsular polysaccharide, using an anti-*S. suis* serotype 2 rabbit serum of ST1 strain P1/7 (*A*) and ST25 strain 89-1591 (*B*). Black bars = 1 µm.

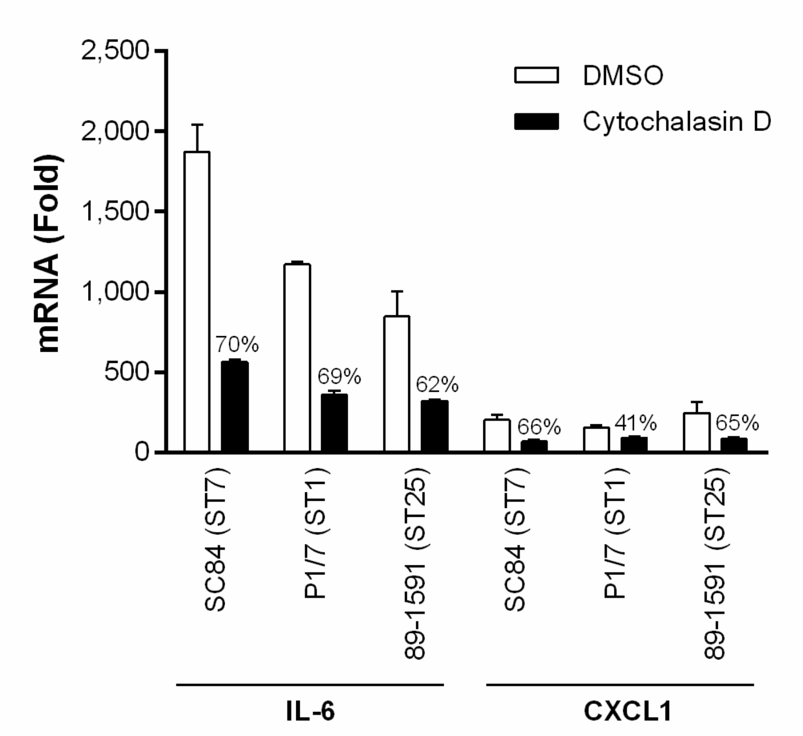

Figure S5. *Streptococcus suis*-induced IL-6 and CXCL1 expression by dendritic cells is partially internalization-dependent. IL-6 and CXCL1 expression by dendritic cells pretreated with cytochalasin D (5 µM) to inhibit actin polymerization 6 h following infection with *S. suis*. Mock-treated cells (DMSO) were used as controls. Data represent the mean ± SEM (n = 3). The percentages of cytochalasin D-mediated inhibition of cytokine expression are indicated.
